# A mathematical model for the effects of amyloid beta on intracellular calcium

**DOI:** 10.1371/journal.pone.0202503

**Published:** 2018-08-22

**Authors:** Joe Latulippe, Derek Lotito, Donovan Murby

**Affiliations:** 1 Mathematics Department, Norwich University, Northfield, Vermont, United States of America; 2 Chemistry and Biochemistry Department, Norwich University, Northfield, Vermont, United States of America; University of Debrecen, HUNGARY

## Abstract

The accumulation of Alzheimer’s disease (AD) associated Amyloid beta (A*β*) oligomers can trigger aberrant intracellular calcium (Ca^2+^) levels by disrupting the intrinsic Ca^2+^ regulatory mechanism within cells. These disruptions can cause changes in homeostasis levels that can have detrimental effects on cell function and survival. Although studies have shown that A*β* can interfere with various Ca^2+^ fluxes, the complexity of these interactions remains elusive. We have constructed a mathematical model that simulates Ca^2+^ patterns under the influence of A*β*. Our simulations shows that A*β* can increase regions of mixed-mode oscillations leading to aberrant signals under various conditions. We investigate how A*β* affects individual flux contributions through inositol triphosphate (IP_3_) receptors, ryanodine receptors, and membrane pores. We demonstrate that controlling for the ryanodine receptor’s maximal kinetic reaction rate may provide a biophysical way of managing aberrant Ca^2+^ signals. The influence of a dynamic model for IP_3_ production is also investigated under various conditions as well as the impact of changes in membrane potential. Our model is one of the first to investigate the effects of A*β* on a variety of cellular mechanisms providing a base modeling scheme from which further studies can draw on to better understand Ca^2+^ regulation in an AD environment.

## Introduction

Alzheimer’s Disease (AD) is a devastating neurodegenerative illness affecting over 40 million people worldwide. AD is the leading cause of dementia and is characterized by a progressive and irreversible decline in memory and cognitive skills [1]. The prevalence of AD and associated dementia is estimated to double in the next 20 years, and as such, there is a critical need to better understand this disease. While the appearance of extracellular hydrophobic amyloid plaques and intracellular neurofibrillar tangles associated with tau proteins have become the hallmarks of the disease, the cause of AD remains unknown. Abnormal intracellular Ca^2+^ levels have been observed in AD brains even before the presentation of clinical symptoms and amyloid plaques [2, 3]. Because it has been shown that Amyloid beta peptide (A*β*) accumulation can lead to increased intracellular Ca^2+^ levels [4, 5], studying its effect on intracellular mechanisms is important for understanding its impact on neuronal functions. Intracellular accumulation of A*β* can cause an increase release of Ca^2+^ from internal stores such as the Endoplasmic Reticulum (ER) [4, 6–8]. As such, A*β* may lead to both local and global Ca^2+^ proliferation that are persistent and cytotoxic. Sustained Ca^2+^ disregulation can trigger apoptosis leading to premature neuronal death, a characteristic feature seen in AD. Although the accumulation of A*β* has been linked to the progression of AD by altering Ca^2+^ signaling processes within neurons and neuroglia, the mechanisms for how and why this occurs are not fully understood.

Our goal is to use mathematical modeling to describe various conditions under which A*β* can lead to aberrant Ca^2+^ signaling. The amyloid hypothesis suggests that the accumulation of A*β* in the brain is the primary driving force of AD pathogenesis [9–12]. In this hypothesis, the formation of A*β* plaques and fibrils are a consequence of the imbalance between the formation and sequestration of A*β*. The slow accumulation of A*β* peptides can alter Ca^2+^ signaling processes leading to synaptic failure and neuronal death. Although it is unclear how A*β* disrupts intracellular Ca^2+^ homeostasis, there is growing evidence that A*β* directly affects the production of inositol triphosphate (IP_3_) [7], calcium-induced calcium release (CICR) through the ryanodine receptor (RyR) [13, 14], and the plasma membrane [15, 16]. We use the results of these works to make simplifying assumptions for how A*β* affects various Ca^2+^ signaling mechanisms in a simplified whole-cell model.

In this study we present a theoretical approach to better understand the driving mechanisms for various Ca^2+^ oscillatory patterns within an AD environment. By developing a mathematical model for intracellular Ca^2+^ regulation, we can begin to study how A*β* affects Ca^2+^ flux through various individual channels and pumps. Investigating model solutions can also provide important information on the impact of A*β* on Ca^2+^ basal levels over various timescales. Due to current experimental limitations, mathematical and computational models can provide insights for targeting specific mechanisms in order to restore neuronal function, and to suggest symptomatic improvement strategies. In fact, according to Liang et al. (2015) there is growing momentum to study Ca^2+^ dynamics in AD, specifically through computational and mathematical modeling, and this work outlines such an approach.

## Methods

### Calcium model formulation

In order to study the effects of A*β* on intracellular Ca^2+^, we first build a simplified whole-cell Ca^2+^ model by making use of the vast array of work on modeling Ca^2+^ dynamics and the calcium signaling “toolkit” (see [17–20] for example). Once this model is developed, we add the influence of A*β* by altering various components of the model. We then analyze model solutions by investigating the dynamical structure for various parameter regimes in order to draw out conditions that lead to changes in basal Ca^2+^ levels and aberrant signals. For our purposes, we characterize aberrant signals as non-periodic oscillations over a timescale of about 100-200 seconds.

We model Ca^2+^ dynamics using traditional methods by tracking the flux in and out of the cytoplasm. Let *c* denote the concentration of free Ca^2+^ ions in the cell cytoplasm, then the rate of change in intracellular Ca^2+^ is governed by

$$\frac{dc}{dt}={\tilde{J}}_{in}-{\tilde{J}}_{out},$$ (1)

where $\tilde{J}$ denotes flux. We assume a spatially homogeneous cell whose volume is fixed and track Ca^2+^ concentration changes in time. As such, the ER and cytoplasm coexist at each point of the cell. Although these simplifying assumptions make the model limited, such an approach has been extremely useful in quantifying and identifying key mechanisms behind certain Ca^2+^ signaling patterns. We take this position to study the influence of A*β* on Ca^2+^ dynamics using a simplified whole-cell model. Many different mathematical approaches have been used to better understand Ca^2+^-mediated neuroglia function (see [21] for an overview of these) and we have utilized this body of work to develop our model below.

**Fig 1:**
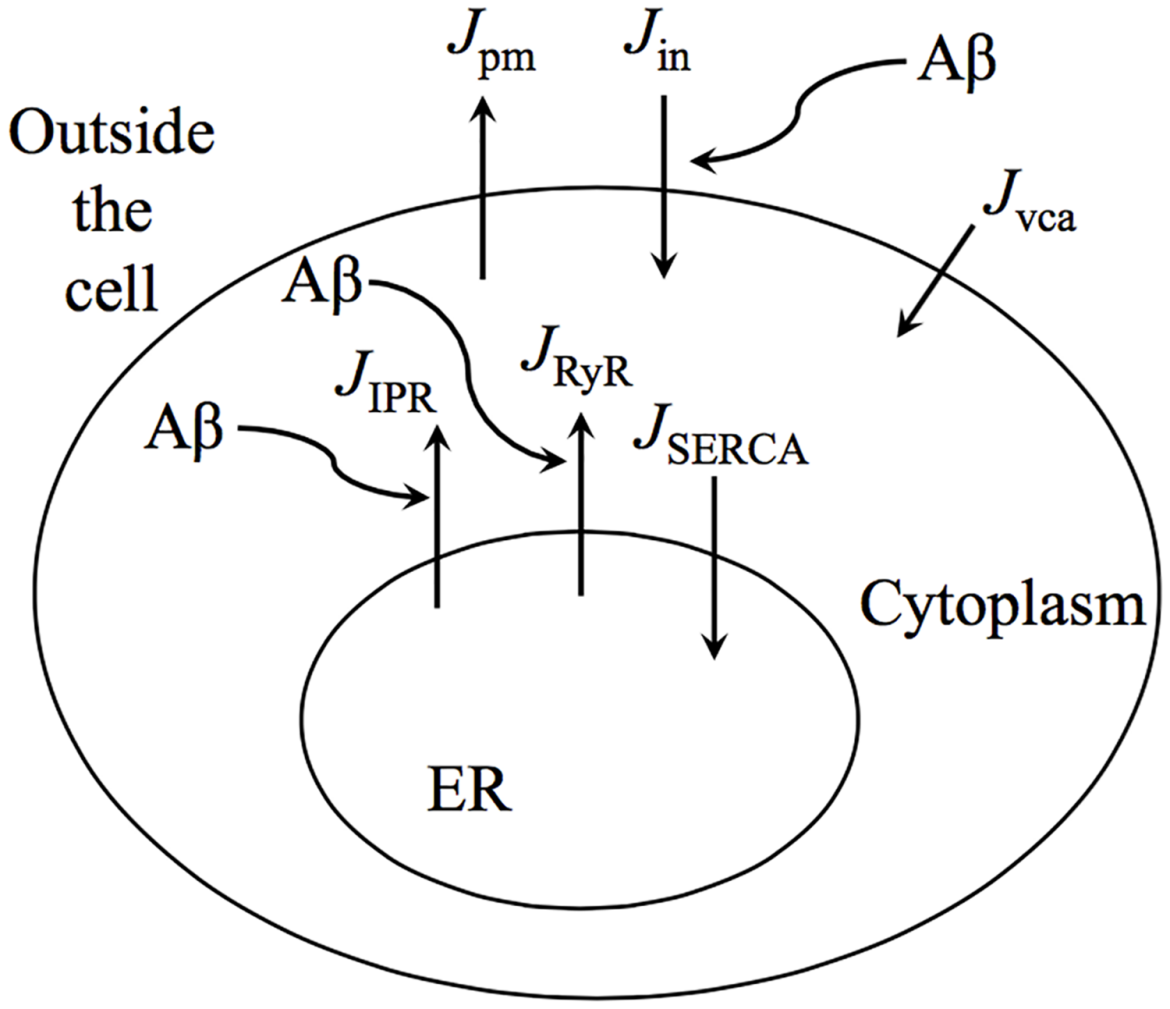
Two pool model diagram. This figure shows the critical flux terms utilized in the formulation of the model. In addition, the relevant fluxes affected by A*β* are highlighted.

Our model structure was selected to account for the major components that have been shown to be influenced by A*β* and that can lead to Ca^2+^ dynamics on the timescale of seconds. We assume that intracellular Ca^2+^ in-fluxes (into the cytoplasm) are those corresponding to IP_3_ receptors (IPR), RyRs, a general membrane leak *J*_*in*_, and we include a fast Voltage Gated Ca^2+^ Channel (VGCC) in *J*_*vca*_ to account for membrane permeability. The out-fluxes (out of the cell or sequestered into Ca^2+^ pools such as the ER) are modeled using a plasma membrane pump *J*_*pm*_, and a sarco-endoplasmic reticulum Ca^2+^ ATPase (SERCA) pump *J*_*SERCA*_. A diagram of the major fluxes outlined in the model is given in Fig 1. Also included in the diagram are the model assumptions of the interaction of A*β* with respect to individual flux term.

We assume that the ER is homogeneously distributed throughout the interior of the cell and that the fluxes of Ca^2+^ through the IPR and RyR are proportional to the difference in the concentration of Ca^2+^ between the ER and the cytoplasm. Under these standard assumptions, using conservation of fluxes, our general Ca^2+^ model takes the form

$$\frac{dc}{dt}=J_{IPR}+J_{RyR}-J_{SERCA}+J_{in}-J_{pm}+J_{vca}$$ (2)

$$\frac{dc_{e}}{dt}=-\gamma (J_{IPR}+J_{RyR}-J_{SERCA})$$ (3)

where *c*_*e*_ denote the concentration of Ca^2+^ in the ER, and *γ* is the ratio of cytoplasmic volume to the ER volume. The individual contributions of each flux can vary from a simple Hill function to more complicated forms involving numerous parameters and additional terms. We provide a description of each of the flux terms we use in our model below. We also provide background information on their development and why it may be well suited for our purposes. Each term was chosen in order to balance meaningful biophysical quantities while maintaining a tractable mathematical structure.

The term *J*_*vca*_ in (2) links membrane potentials with other Ca^2+^ signaling mechanisms. Ca^2+^ regulation and changes in membrane potential do depend on each other and a model that connects their influence may be critical for advancing our understanding of the long term effects of A*β* on Ca^2+^ signaling. To address this, we include a section in our results that incorporates the effects of membrane potentials into our model and provide some examples of the impact of A*β* on the dynamics. Although a full exploration of the role of membrane potential on Ca^2+^ dynamics is beyond the scope of the current study, we do provide the base structure from which to build a more complete whole-cell model.

Ca^2+^ regulation in non-excitable cells, such as astrocytes, is extremely complex and influenced not only by membrane potential but also Ca^2+^ buffering. Cytoplasmic Ca^2+^ buffering plays a significant role in calcium’s ability to move throughout the cell. Almost all of the available Ca^2+^ is bound to buffers and free cytoplasmic Ca^2+^ cannot move very far before being bound [18]). Because Ca^2+^ diffusion plays an important role in spatiotemporal signaling, nonlinear buffering may provide some insights behind certain types of oscillatory patterns and Ca^2+^ waves. However, compared to the temporal release and uptake of Ca^2+^ into internal stores, buffering occurs on a much faster timescale. In our model we have assumed that Ca^2+^ buffering is fast, immobile, and has low affinity. Using these assumptions, we have scaled the model to account for fast and linear buffers even though an explicit description of buffering is not provided (further details on buffering can be found in [18]).

#### IP_3_ receptor model

IP_3_ is a ligand produced by phospholipase C (PLC) when activated by a G-protein coupled receptor reacting to an external stimulus such as neurotransmitters, hormones, and growth factors [22]. As a key secondary messenger, IP_3_ regulates many important cellular functions, including the release of Ca^2+^ from the ER through the IP_3_ receptor [23]. We assume that the flux from the IP_3_ receptor follows a saturating binding rate model of the form found in [24, 25]. Thus, we write

$$J_{IPR}=\left(k_{f}P_{0}+J_{er}\right)\left(c_{e}-c\right)$$ (4)

where *k*_*f*_ controls the density of the IP_3_ receptor, *J*_*er*_ is a leak from the ER to the cytoplasm, and *P*_0_ is the open probability of the IPR. The leak term is necessary to balance the ATPase flux at steady state. To model *P*_0_, we use a version of the Sneyd and Dufour (2002) model that is based on previous models found in [26–28]. The model assumes that the receptor can be in one of six states, with *R* the resting state, *O* the open state, *A* the active state, *S* the shut state, and *I*_1_ and *I*_2_ both represent inactive states. The transitions between states can depend on Ca^2+^ and IP_3_ concentration *p*. As such, the IP_3_ receptor will be in various states depending on the value of *p*.

In this model, the receptor open probability is given by

$$P_{0}={\left(\alpha_{1}O+\alpha_{2}A\right)}^{4}$$ (5)

where *α*_1_ and *α*_2_ are parameters that control the individual contributions of the open and active states of the receptor. As in [24], we assume that *α*_1_ = 0.1 and *α*_2_ = 0.9. In the model, it is assumed that the receptor has four identical and independent subunits and that Ca^2+^ flows when all four subunits are in either the *O* or *A* state. The equations for the transitions between the states are given in [24], but have also been included in the Supplementary Appendix for completeness. This model was chosen since it does respond reasonably well to changes in Ca^2+^ concentration and IP_3_ concentrations [25].

#### RyR model

To model the contribution of the RyR, we utilize the algebraic model of Friel (1995). The receptor is modeled as a simple leak channel, with a flux through the channel proportional to the concentration difference between the ER and cytosol. This model was used to investigate Ca^2+^ oscillations in a sympathetic neuron. Thus, the flux through the RyR is given by

$$J_{RyR}=k_{3}\left(c_{e}-c\right).$$ (6)

To more accurately model CICR, the rate constant *k*_3_ is defined as an increasing sigmoidal function of intracellular Ca^2+^ concentration and takes the form

$$k_{3}=k_{1}+\frac{k_{2}c^{n}}{k_{d}^{n}+c^{n}},$$ (7)

where *k*_1_, *k*_2_, and *k*_*d*_ are parameters. We use *n* = 3 to match the experimental results obtained in [29]. The parameter *k*_1_ in (7) is the zero calcium concentration level leak. This term is often used to ensure a physiologically significant resting Ca^2+^ level [18]. The term *k*_*d*_ corresponds to the RyR channel sensitivity for CICR, and *k*_2_ is the maximal rate of the channel. The simple nature of the Friel model makes it a viable choice especially since data for the contributions of Ca^2+^ flux through the RyR in the presence of A*β* are minimal.

#### Leak, membrane pump, and SERCA

The membrane leak *J*_*in*_ is modeled using a linearly increasing function of IP_3_ concentration [30]. Although this increase may be due to various mechanisms, here we only include a linearly increasing contribution to make sure that steady-state Ca^2+^ concentration depend on *p*. Thus,

$$J_{in}=a_{1}+a_{2}p,$$ (8)

where *a*_1_ and *a*_2_ are parameters.

When modeling Ca^2+^ dynamics both the SERCA pump and the plasma membrane pump play an important role in maintaining concentration gradients. Different models of these pumps exist and vary in complexity. Here we model the plasma membrane pump using a simple Hill equation of the form

$$J_{pm}=\frac{V_{pm}c^{n}}{K_{pm}^{n}+c^{n}},$$ (9)

where *V*_*pm*_ is the maximal velocity and *K*_*pm*_ is the channel sensitivity. We have chosen a standard Hill coefficient of 2 as is found in [31].

To describe the behavior of the SERCA pump we utilize a simplified four state bidirectional Markov model (as found in [20]) of the form

$$J_{serca}=\frac{c-K_{1}c_{e}}{K_{2}+K_{3}c+K_{4}c_{e}+K_{5}cc_{e}}$$ (10)

to model the SERCA pump. In (10), *K*_*i*_ for *i* = 1, …, 5 are constants. Notice that this choice of model is more complicated than a simple Hill equation. Experimental evidence show that the rate of the pump may be modulated by the level of Ca^2+^ in the ER [32, 33], and this model provides a way to account for both *c* and *c*_*e*_.

#### Voltage dependent calcium channel

The flux due to changes in membrane potential is given in our model by *J*_*vca*_. When the volume of the cell is constant, Ca^2+^ flux and current are related by the equation

$$J_{vca}=\frac{-I_{ca}}{2Fw},$$ (11)

where *I*_*ca*_ is the current through the VGCC, *F* is Faraday’s constant, and *w* is the cell volume. Since *I*_*ca*_ depends on the membrane potential *V* and Ca^2+^, we need a way to track membrane potential. Here, we assume that the membrane potential follows a Hodgkin and Huxley formulation [34] that satisfies

$$C_{m}\frac{dV}{dt}=-\sum I_{x},$$ (12)

where *C*_*m*_ is the cell capacitance, *V* is the membrane potential, and the sum is over all ionic currents across the cell membrane. In the case of non-excitable astrocytes, current flow across its membrane is characterized by potassium (K^+^)-selective membrane conductance [35] and voltage dependent channels [36, 37]. As such, our membrane equation takes the form

$$C_{m}\frac{dV}{dt}=-I_{kir}\left(V\right)-I_{na}\left(V\right)-I_{l}\left(V\right)-I_{ca}\left(V\right)+I_{app},$$ (13)

where *I*_*kir*_, *I*_*na*_, *I*_*l*_, and *I*_*ca*_ correspond to an inward rectifying potassium, sodium, leak, and Ca^2+^ current, respectively, and *I*_*app*_ is an applied current. In (13), we utilize a formulation for *I*_*kir*_(*V*) similar to [38] and write

$$I_{kir}\left(V\right)=-g_{kir}(\frac{\sqrt{K_{0}}}{1+\text{exp}(\left(V-V_{ka}-V_{a2}\right)/V_{a3})})\left(V-V_{ka}-V_{a1}\right),$$ (14)

where *g*_*kir*_ is the maximal channel conductivity, *V*_*ka*_ is the Nernst K^+^ potential, *K*_0_ is the extracellular K^+^ concentration, and *V*_*a*1_, *V*_*a*2_ and *V*_*a*3_ are constants. In the model of [38], *K*_0_ is time dependent and depends on proximal neuronal activity (further details of this formulation can be found in [38]). Here, we simplify this and assume that the external compartment is saturated with K^+^ and as such consider *K*_0_ to be constant. Each of the remaining currents have the form *I*_*x*_ = *g*_*x*_(*V* − *V*_*x*_), where *g*_*x*_ is the conductance and *V*_*x*_ is the Nernst potential of each channel.

Although many alternative formulations for *I*_*ca*_ exist (such as the Goldman-Hodgkin-Katz current approximation), we use a T-type like channel (as that found in [39]) of the form

$$I_{ca}\left(V\right)={\bar{g}}_{caT}m_{caT}^{2}h_{caT}\left(V-V_{ca}\right),$$ (15)

where ${\bar{g}}_{caT}$ is the maximal conductance, *V*_*ca*_ is the Ca^2+^ Nernst potential, and *m*_*caT*_ and *h*_*caT*_ are gating variables similar to those used for the Na^+^ channel in the Hodgkin and Huxley model. A T-type Ca^2+^ current has a low-threshold activation with an inactivating gating variable that exhibit sub-threshold oscillations and inhibitory rebound bursts. Although other types of Ca^2+^ currents (such as L-type, Ca^2+^-dependent potassium channels, etc.) can also be included in the model, here we simply illustrate how one can link membrane potential with other Ca^2+^ regulatory mechanisms together into a single model and use that model to investigate the role of A*β* on Ca^2+^ dynamics. As such, the T-type Ca^2+^ channel described in (15) is sufficient for studying how changes in membrane potentials could impact intracellular Ca^2+^. Full details of the membrane potential model are provided in the Supplementary Appendix.

### Amyloid beta assumptions

The influence of A*β* on individual pumps, channels, and exchangers remains largely unknown. However, some studies provide insight on the effects of A*β* on Ca^2+^ regulation and we utilize these findings in making our assumptions about the effects of A*β*. For example, De Caluwé and Dupont (2013) formalized a theoretical model to describe a possible feedback loop between Ca^2+^ and A*β*. Their model showed that the existence of a bistable steady-state region can exists in the presence A*β*. The model suggests that over time, cytosolic Ca^2+^ proliferation as a result of A*β* could trigger the onset of AD. In this study, we are interested in the generation of aberrant Ca^2+^ signals on a relatively short timescale. Since the accumulation of A*β* can occur over months, years, and even decades, A*β* concentrations changes occur on a very long timescale compared to changes in Ca^2+^. As such, we assume that A*β* may be present in the environment, but make no attempt to track changes in A*β* concentration over time.

The formation of A*β* plasma membrane pores can alter Ca^2+^ signaling by creating additional influx into the cytoplasm [15, 16, 40, 41]. In order to incorporate the possible influence of A*β* generated plasma membrane pores, we use a similar mechanism as found in [42]. Let *a* represent a fixed level of A*β* concentration present in the environment. Then, we include the term *k*_*β*_ *a*^*m*^ in *J*_*in*_ and write an altered membrane leak flux as

$$J_{in}=a_{1}+a_{2}p+k_{\beta }a^{m},$$ (16)

where *m* represents a cooperativity coefficient, and *k*_*β*_ is a rate constant (see [42] for details).

Although it is well known that A*β* can disrupt RyR-regulated Ca^2+^ signals, the mechanisms for how this happens remains controversial [43–45]. Several studies have addressed the role of RyR-regulated Ca^2+^ disruptions in AD [46–49]. Paula Lima et al. (2011) report that A*β* can generate prolonged Ca^2+^ signals *in vitro* through RyR in primary hippocampal neurons of rat embryos as a result of NMDAR-dependent Ca^2+^ entry through the plasma membrane. These results agree with studies showing that A*β* can cause substantial Ca^2+^ influx through NMDAR [50, 51]. In our model these types of fluxes are combined and accounted for in *J*_*in*_, although in a somewhat crude fashion. Futhermore, A*β* can increase RyR channel open probability on a short timescale [4, 14]. In order to account for this, we alter the RyR channel sensitivity term and assume that it is affected by A*β*. Thus, our altered RyR model takes the form

$$k_{3}=k_{1}+\frac{k_{2}c^{n}}{{\left(k_{d}+k_{\alpha }a\right)}^{n}+c^{n}},$$ (17)

where *k*_*α*_ is positive and controls the strength of the influence of A*β*. Notice that an increase in *a* corresponds to an increase in the RyR sensitivity.

#### Calcium model formulation with amyloid beta

The simplified whole-cell Ca^2+^ model described above tracks changes in cytosolic Ca^2+^ concentration as a function of time in the presence of A*β*. By putting the different channels, exchangers, and pumps together, our Ca^2+^ model takes the form

$$\begin{array}{ccc} \frac{dc}{dt} & = & (k_{f}{\left(0.1O+0.9A\right)}^{4}+k_{1}+\frac{k_{2}c^{3}}{{\left(k_{d}+k_{\alpha }a\right)}^{3}+c^{3}})\left(c_{e}-c\right)\\ & - & \frac{c-K_{1}c_{e}}{K_{2}+K_{3}c+K_{4}c_{e}+K_{5}cc_{e}}+\left(a_{1}+a_{2}p+k_{\beta }a^{m}\right)-\frac{V_{pm}c^{2}}{K_{pm}^{2}+c^{2}}\\ & - & p_{s}{\bar{g}}_{caT}m_{caT}^{2}h_{caT}\left(V-V_{ca}\right), \end{array}$$ (18)

$$\begin{array}{ccc} \frac{dc_{e}}{dt} & = & -\gamma [(k_{f}{\left(0.1O+0.9A\right)}^{4}+k_{1}+\frac{k_{2}c^{3}}{{\left(k_{d}+k_{\alpha }a\right)}^{3}+c^{3}})\left(c_{e}-c\right)\\ & - & \frac{c-K_{1}c_{e}}{K_{2}+K_{3}c+K_{4}c_{e}+K_{5}cc_{e}}]. \end{array}$$ (19)

Notice that this model has twelve dynamic equations, with five of those controlling the IP_3_ receptor (*R*, *O*, *A*, *I*_1_, and *I*_2_), three controlling the membrane potential (*V*, *m*, and *h*), and an additional two variables controlling the gating variables *m*_*caT*_ and *h*_*caT*_ (a description of these additional equations are provided in the Supplementary Appendix). We have also added a scaling and control parameter *p*_*s*_ to simplify the contribution from the VGCC flux. When *p*_*s*_ = 0, the impact of membrane potential on Ca^2+^ signaling are ignored. When *p*_*s*_ > 0, changes in membrane potential do influence the dynamics of intracellular Ca^2+^. The large set of parameter values was selected to closely match those of previous studies whenever possible and can be found in Table 1.

**Table 1:** Parameter values of the Ca^2+^ model (18) and (19).

Parameters			
*k*_*f*_	0.98 s^−1^	*γ*	5.4
RyR			
*k*_1_	0.013 s^−1^	*k*_*d*_	0.13 *μ*M
*k*_2_	0.18 s^−1^	*k*_*α*_	.75
SERCA			
*K*_1_	0.0001	*K*_2_	0.007 s
*K*_3_	0.06 *μ*M^−1^ s	*K*_4_	0.0014 *μ*M^−1^ s
*K*_5_	0.007 *μ*M^−2^ s		
Transport			
*a*_1_	0.003 *μ*M s^−1^	*k*_*β*_	1 s^−1^
*a*_2_	0.02 s^−1^	*m*	4
*V*_*pm*_	2.8 *μ*M s^−1^	*K*_*pm*_	0.425 *μ*M
K^+^ Channel			
*g*_*kir*_	60 pS	*K*_0_	2 mM
*V*_*a*1_	-14.83 mV	*V*_*a*2_	34 mV
*V*_*a*3_	19.23 mV	*V*_*ka*_	-65.2 mV
VGCC			
${\bar{g}}_{caT}$	.45 mS ⋅ ms^−1^	*V*_*ca*_	100 mV

In the formulation of our model we have assumed that the presence of A*β* can increase membrane permeability by creating plasma membrane A*β* pores, and have used the work of [42] to alter our membrane in-flux. Additionally, we have assumed that the presence of A*β* increases RyR sensitivity and have incorporated additional terms in the RyR component of the model. Currently in our model, IP_3_ acts as the primary agonist that can then trigger Ca^2+^ release from IP_3_ receptors. In addition to affecting the IP_3_ receptor open probability, Ca^2+^ release activates RyR and subsequent CICR. In the next chapter we separate our analysis for the model under three assumptions. We first characterize the solutions of the model for constant levels of IP_3_ and when the effects of membrane potential are ignored. Second, also in the absence of membrane potentials, we expand our model to include a dynamic variable for IP_3_ production. Lastly, we include the effects of membrane potential on Ca^2+^ dynamics by incorporating flux through VGCC under various levels of fixed IP_3_. We further look to characterize the impact of VGCC on model solutions in the presence of A*β*.

Numerical solutions of the differential equations systems were obtained using explicit Runge-Kutta (4,5) algorithms implemented in Matlab [52]. Bifurcation analyses were done using the AUTO [53] extension of XPPAUT [54]. AUTO is a software program used to analyze the bifurcation structures of systems of ordinary differential equations such as (18) and (19). In the bifurcation diagram illustrated in this manuscript, the bifurcation parameter is plotted on the *x*-axis and the projection of stable and unstable steady-state solutions, upper and lower branches of periodic orbits, and critical transitions points (such as Hopf and period doubling) are labeled accordingly. Hopf bifurcations occur when a steady-state solution loses it’s stability as a result of small changes in a parameter. Mathematically, this occurs when complex eigenvalues (of the linearization) cross the imaginary axis away from the origin. Period doubling bifurcations occur when a small change in a bifurcation parameter causes the period of an oscillatory system to double. These and other types of bifurcations can be used to describe systemic changes in dynamics and in our case, transitions from single-mode oscillations to mixed-mode oscillations (MMOs). The program AUTO allows us to readily compute the location of these bifurcations as model parameters are varied. Initial conditions for all simulations were set at *c*_0_ = 0.05 and *c*_*e*_ = 10 with *R*_0_ = 1 and all other IP_3_ receptor initial conditions are set to zero. Initial conditions for the membrane potential are *V*_0_ = −65, *m*_0_ = 0.05, *n*_0_ = 0.32, *h*_0_ = 0.6, *m*_*caT*0_ = 0.29, and *h*_*caT*0_ = 0.01.

## Results

In all three sections below, the model dynamics show that aberrant Ca^2+^ can emerge in the presence of A*β*. These aberrant signals can occur under various conditions suggesting a complex link between the influence of A*β* and the model components. As such, we break down the dynamics of the model by tracking solutions as a result of altering one or two parameters within a specific signaling component. We first look at model solutions when IP_3_ concentration is fixed and persists and is not influenced by membrane potential. We then allow for IP_3_ to change dynamically and simulate the response in Ca^2+^ with and without the influence of A*β*. Lastly, we incorporate the influence of membrane potentials and investigate model solutions for various levels of A*β* when IP_3_ concentration is fixed.

### Calcium model with constant IP_3_ and no membrane potential

Simulations of (18) and (19) with *p*_*s*_ = 0 show that incorporating A*β* in a simplified Ca^2+^ model can lead to aberrant Ca^2+^ signaling through dynamical transitions into MMOs. Particularly, we look to track the influence of A*β* on the location of Hopf points (labeled HB), period doubling points (labeled PD), and regions of MMOs (shaded) in order to better understand Ca^2+^ steady-states and oscillatory patterns. From a phenomenological perspective, a transition through MMO may explain why A*β* can trigger persistent aberrant Ca^2+^ signals. Although the long-term accumulation and influence of A*β* remains difficult to model, our approach illustrates that aberrant Ca^2+^ signals can occur under the influence of A*β* for a variety of parameter regimes.

#### IP_3_ influence on model dynamics

Experimentally, IP_3_ can be photoreleased simultaneously through out a cell. In such conditions, IP_3_ diffusion is minimized and can be treated as constant. By varying the amount of IP_3_ available in the cytosol, the model can exhibit Ca^2+^ oscillations as typically found in various cell types. These oscillatory patterns are critical in order for cells to maintain appropriate concentration gradients and to re-establish homeostasis levels after a triggering event. In the absence of A*β*, model Ca^2+^ oscillations appear and disappear as a result of transitions through Hopf bifurcations as the parameter *p* increases. Although these stable oscillatory patterns are predictable for a large domain of the parameter *p*, the model does exhibit MMOs for small parameter regions near the Hopf points. It is these MMO patterns that we are most interested in. Our results show that in the presence of A*β*, the regions of MMOs can grow and become larger. This leads to an increased possibility for aberrant Ca^2+^ signals. We hypothesize that aberrant Ca^2+^ signals observed experimentally can be described as dynamic transitions through MMOs. The complicated patterns in MMOs are often characterized by sub-threshold oscillations interspersed within large relaxation types of oscillations [55–57].

**Fig 2:**
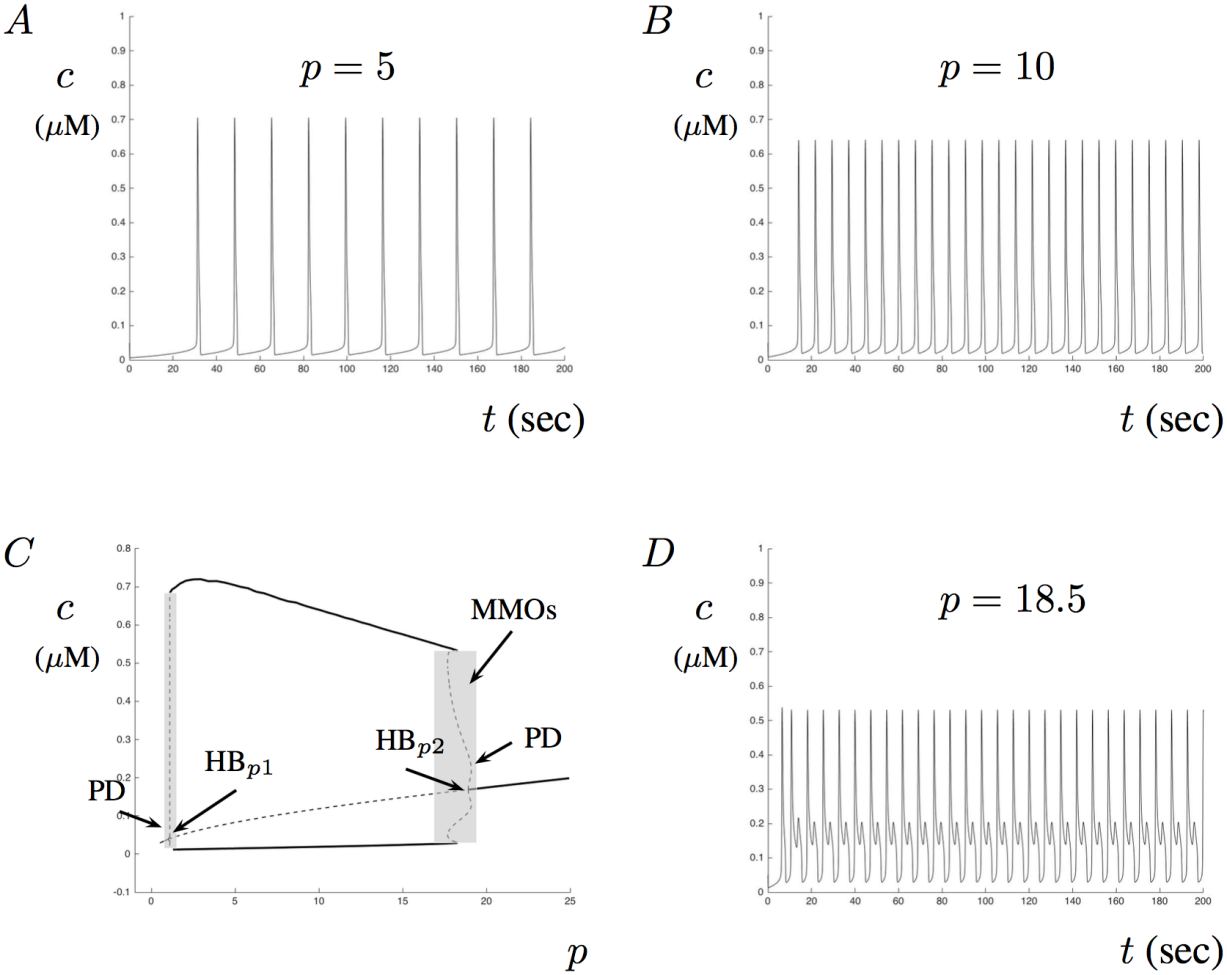
Calcium dynamics for constant IP_3_ levels. Properties of the Ca^2+^ model when IP_3_ concentrations are fixed and no A*β* is present (i.e., *a* = 0). A and B show the numerical solution with *p* = 5 and *p* = 10, respectively. For these parameter values, the frequency of oscillations increases as *p* increases. C illustrates the partial bifurcation diagram for the model with *p* as the bifurcation parameter. The middle curve crossing the diagram correspond to the steady-state values (solid for stable, dashed for unstable). Also shown are the maximum and minimum amplitudes of the periodic orbits of the model (solid curves above and below the steady-state curve). Key bifurcation points are labeled HB_*p*1_ and HB_*p*2_ (Hopf), and PD (period-doubling). The shaded area corresponds to the regions where MMOs are present. D shows MMOs for *p* = 18.5.

Illustrated in Fig 2A and 2B are numerical simulations of the model, with *a* = 0 that exhibit sustained Ca^2+^ oscillations as the amount of *p* is increased from *p* = 5 to *p* = 10. An increase in frequency occurs as *p* increases within the predictable region and can be seen by comparing Fig 2A and 2B. In Fig 2C, a partial bifurcation diagram showing the stable maximum and minimum periodic amplitudes is given along with the regions of MMOs and the critical transitions points. Fig 2D shows the solution of the model for *p* = 18.5. For this *p*-value, the model undergoes MMOs with varying oscillatory patterns of mixed amplitude, but we do not characterize it as aberrant Ca^2+^ signaling since it is periodic. Notice that no Ca^2+^ oscillations occur for small and large values of *p*. This is due to the assumption that the IP_3_ receptor is unlikely to open at high and low concentrations of IP_3_. Although our model does not exhibit a typical Hopf bubble, it has the overall qualitative patterns of a robust Ca^2+^ model as described in [18].

#### The presence of amyloid beta

We consider an AD environment where some accumulation of A*β* has occurred but assume that this amount is fixed during the timescale of our simulations. As such, we assume that *a* is constant and that the value correlates to the concentration of A*β* in *μ*M. That is, the value of *a* in the model may be thought of as reflecting the stage of the progression of the disease. For example, a small value of *a* corresponds to a small amount of A*β* that may have accumulated in the early stages of AD, while a larger value of *a* may correspond to a late stage AD. As the parameter *a* is varied, we can then study how the dynamics of the model change as the level of A*β* changes. Our ultimate goal is to better understand the implications of various levels of A*β* on Ca^2+^ so that we may gain knowledge about the possible progression of AD.

#### The effects of A*β* on calcium steady-state levels

The accumulation of A*β* can lead to increased steady-state levels as well as large amplitude oscillations even in the absence of IP_3_ signaling. To better understand the effects of A*β* on model solutions, we simulate the model and look at the effects of changing the parameter *a* on steady-state levels. Various experimental studies have used A*β* levels of 0.5 and 1.0 [13, 50, 51]. As such, we consider a range of values for *a* that matches these types of levels.

**Fig 3:**
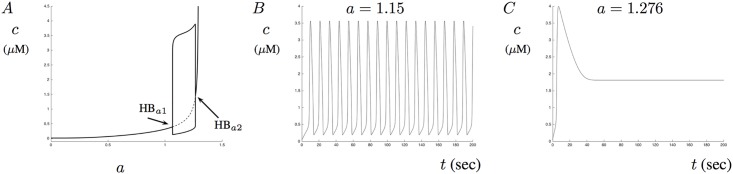
The effects of A*β* on steady-state levels. This figure shows the effects of A*β* on the steady-state Ca^2+^ levels in the absence of IP_3_. A shows a bifurcation diagram with *a* as the bifurcation parameter. Two Hopf bifurcations, labeled HB_*a*1_ and HB_*a*2_, give rise to a Hopf bubble with relatively large oscillation amplitude. The steady-state level quickly becomes unphysical as the amount of A*β* increases towards *a* = 1.3. B shows stable Ca^2+^ oscillations for *a* = 1.15. C shows the solution when *a* = 1.276.

In the case when no IP_3_ is present, the accumulation of A*β* increases the steady-state of Ca^2+^ and can even lead to large-amplitude Ca^2+^ oscillations. Illustrated in Fig 3A is a bifurcation diagram generated by changing the parameter *a* when *p* = 0. Notice that the steady-state value slowly increases as *a* increases until solutions transition into stable periodic oscillations between the two Hopf points HB_*a*1_ and HB_*a*2_. As *a* increases towards the value of 1.293, the steady-state asymptotes and solutions quickly become non-physical. Fig 3B shows a solution where large amplitude oscillations exist for *a* = 1.15. Fig 3C shows the solution for *a* = 1.276. Notice that in this case, the solution first undergoes a large Ca^2+^ increase before settling in on a steady-state value. When the model is presented with levels of A*β* greater than *a* = 1.29, solutions become intractable and are not analyzed.

#### Two Parameter Analysis

**Fig 4:**
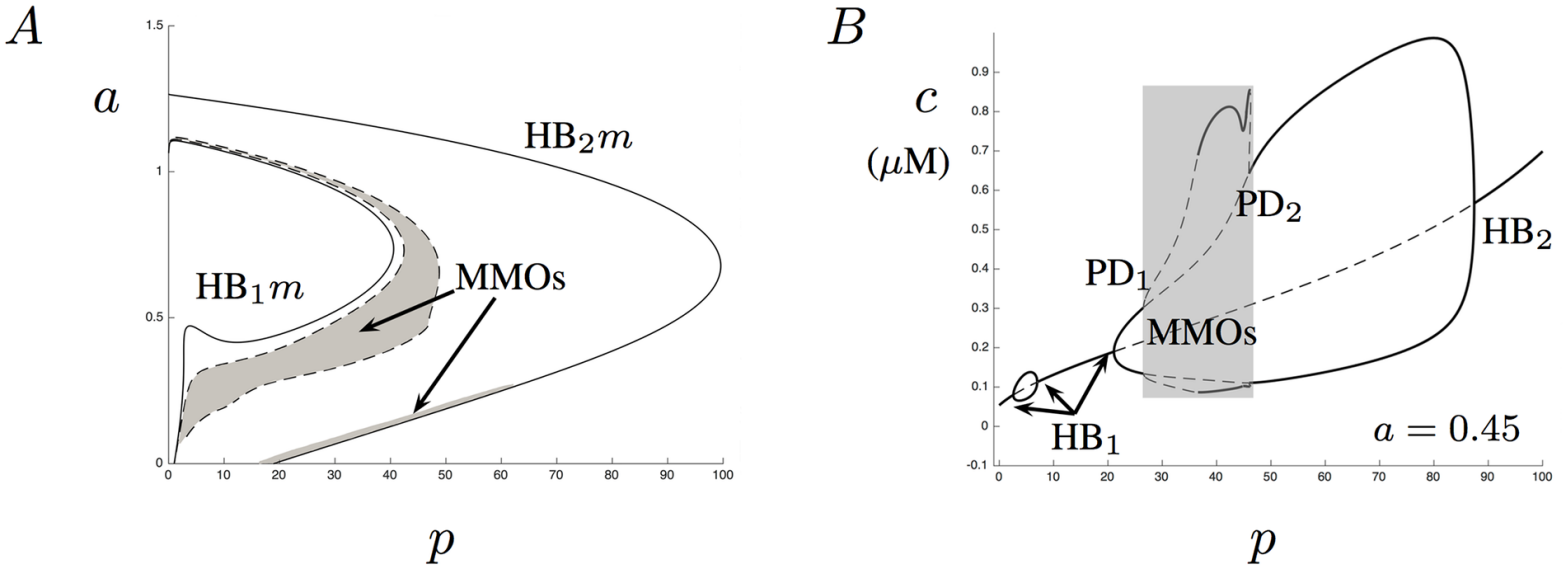
Two parameter bifurcation. A shows a two parameter bifurcation diagram when *p* and *a* are varied. The solid curves in the diagram correspond to the Hopf bifurcation manifolds and are labeled HB_1*m*_ and HB_2*m*_. The dashed lines correspond to manifolds of period doubling points. The shaded regions (between the dashed lines and near the bottom of HB_2_ *m*) correspond to regions where MMOs occur. B shows a bifurcation diagram for *a* = 0.45. Notice that for this value of *a*, four Hopf bifurcations exist and three of them have been labeled with HB_1_ while the last is labeled HB_2_. This figure also illustrates the intermediate region of MMOs between the labels PD_1_ and PD_2_.

Since both *a* and *p* can affect the dynamics of the model, we look at their effects together. Given in Fig 4A is a partial two-parameter bifurcation set for the whole-cell model (18) and (19) when *p*_*s*_ = 0 using *p* and *a* as the bifurcation parameters. The solid lines correspond to the Hopf bifurcation manifolds, while the dashed lines correspond to the period-doubling manifolds. The shaded area in Fig 4A corresponds to the parameter values that elicit MMOs. Notice that in addition to the large MMO region between the dashed lines, there is a small thin region near the Hopf bifurcation manifold labeled HB_2_ *m*. These MMOs vanish for values of *a* > 0.275. This bifurcation diagram has a number of interesting features that can bring to light important behavior in Ca^2+^ regulation. For example, when 0.415 < *a* < 0.472 the bifurcation diagram has four Hopf points. This leads to small amplitude oscillations for certain ranges in the parameter *p*. Fig 4B shows the bifurcation diagram when *a* = 0.45. In this diagram, three of the Hopf bifurcations have been labeled as HB_1_ since all three points lie on the Hopf manifold labeled HB_1_*m* in Fig 4A.

**Fig 5:**
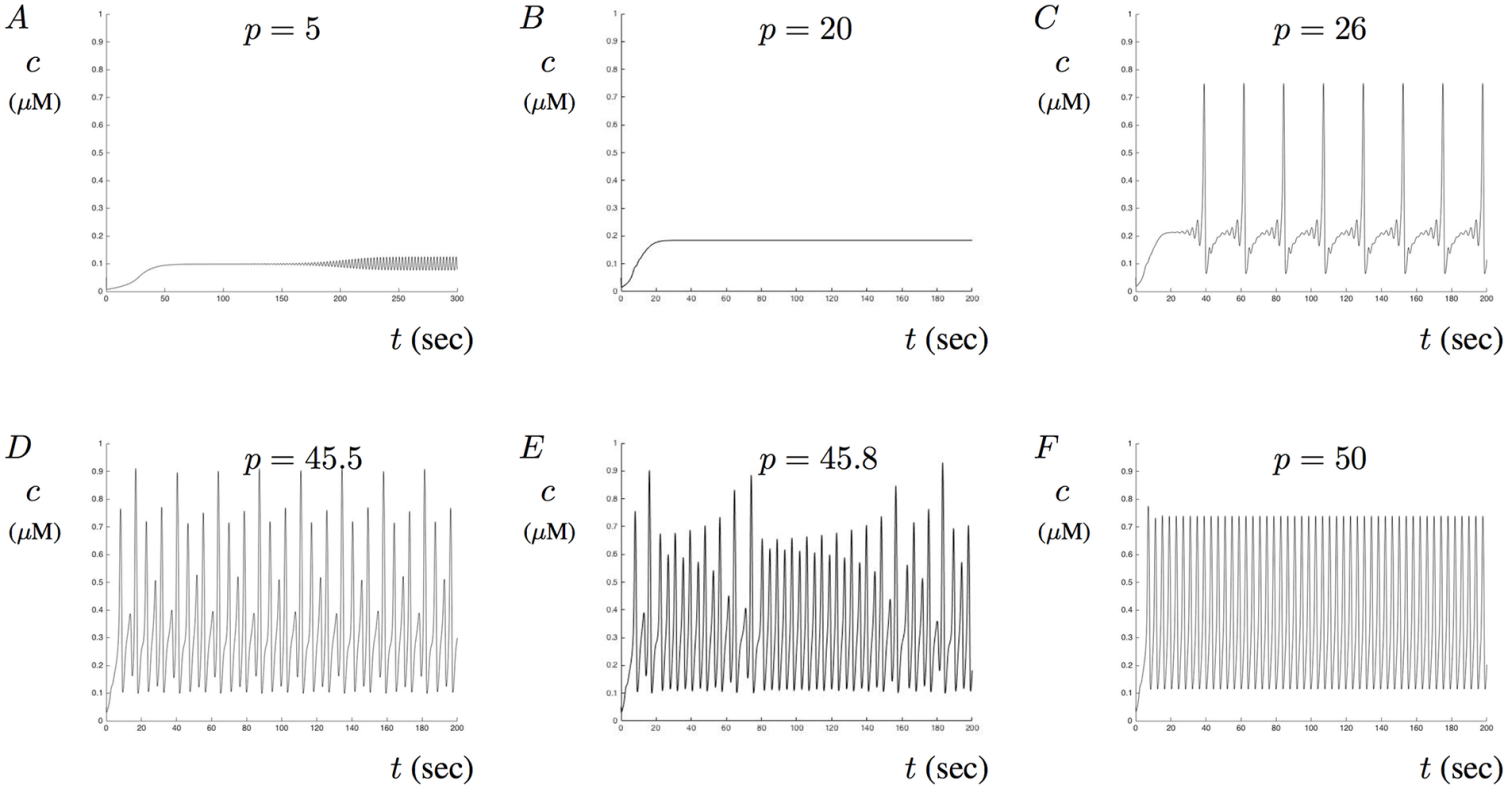
Calcium oscillations in the presence of A*β*. This figure shows various solutions when *a* = 0.45. A shows small amplitude oscillations when *p* = 5 while B shows a stable-steady state solution when *p* = 20. C and D both show MMOs with multiple sub-threshold oscillations when *p* = 26 and *p* = 45.5, respectively. E shows aberrant Ca^2+^ signals when *p* = 45.8. F shows sustained Ca^2+^ oscillations when *p* = 50.

To help us understand this bifurcation structure, various solutions are plotted in Fig 5 and compared to the bifurcation structure given in Fig 4. In Fig 5, *a* = 0.45 is kept fixed while various values of *p* are used. The corresponding model solutions show different types of behaviors ranging from steady-state Ca^2+^ levels, stable periodic solutions, aberrant Ca^2+^ signals, and MMOs. Illustrated in Fig 5A is solution that exhibits small amplitude oscillations with *p* = 5. This solution corresponds to the region where the small Hopf bubble exists as shown in Fig 4B. Fig 5B shows a solution that reaches a stable steady-state when *p* = 20. Fig 5C and 5D show two different solutions that exhibit MMOs for *p* = 26 and *p* = 45.5, respectively. Fig 5D shows aberrant Ca^2+^ signals when *p* = 45.8. Notice that this solution enters aberrant oscillations as the bifurcation structure transitions through period doubling points within the MMO region. Fig 5D shows sustained Ca^2+^ oscillations when *p* = 50.

**Fig 6:**
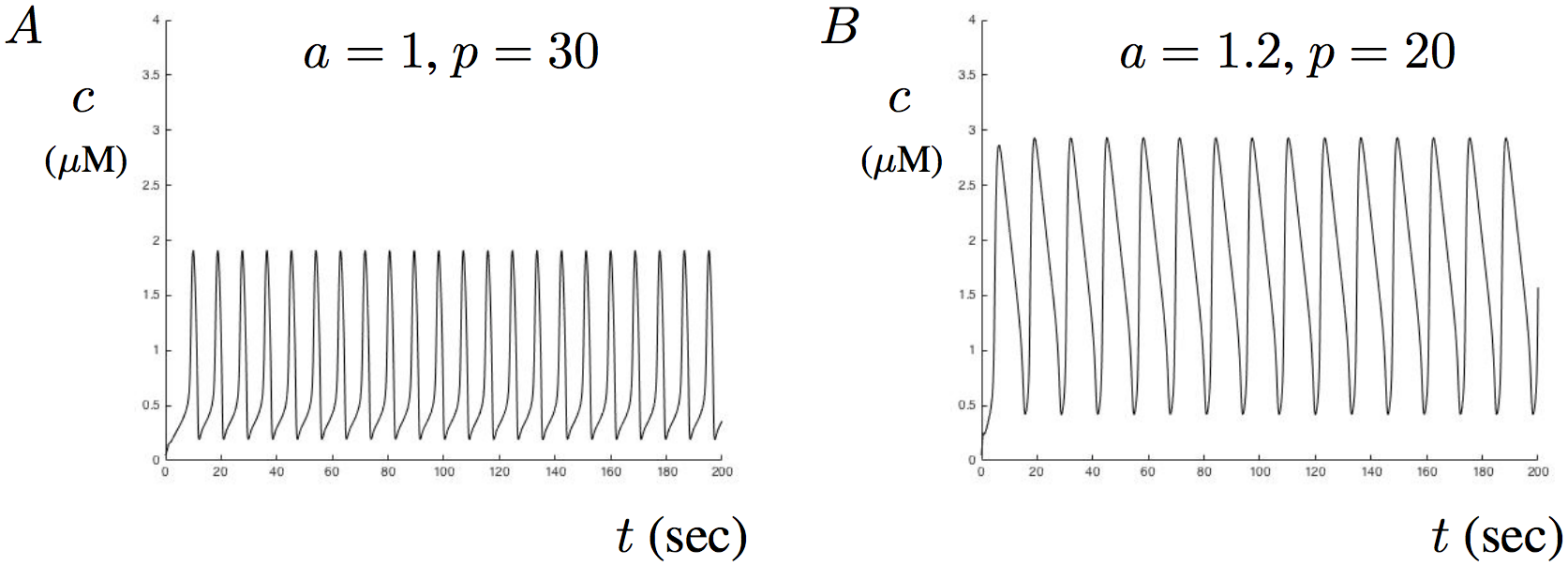
Effects of *a* and *p* on Ca^2+^ oscillation amplitudes. This figures shows two examples of sustained Ca^2+^ oscillations for two sets of parameter selections of *a* and *p*. A shows Ca^2+^ oscillations with peak amplitudes around 2 corresponding to *a* = 1 and *p* = 30. B shows similar oscillations with peak amplitude closer to 3 corresponding to *a* = 1.2 and *p* = 20.

The results of the two parameter bifurcation analysis seem to suggest that A*β* not only increases the steady-state level, but also influences the regions of MMOs. Particularly, the internal range where MMOs emerge between the Hopf manifolds increases for a large parameter range of *a*. As such, the model suggests that A*β* may drive aberrant Ca^2+^ signals as transitions through MMOs. However, as the simulated amount of A*β* increases towards *a* = 1, the region of MMOs decreases and stable periodic orbits exist for most of the *p* parameter range. This condition is illustrated in Fig 6 where two solutions are shown for relatively large values of *a*. Fig 6A shows the solution for *a* = 1 with *p* = 30, while B shows the solution for *a* = 1.2 and *p* = 20. In these cases, Ca^2+^ oscillations have much larger amplitudes than in the absence of A*β*.

#### Contribution of A*β* on calcium signaling through the ryanodine receptor

To better understand the influence of A*β* on Ca^2+^ signaling, we now turn our attention to the contributions of the RyR. Recall that in our model we assume that A*β* affects the RyR by altering the receptor’s sensitivity for CICR. To determine the specific contribution of this altered sensitivity, we first simulate the model for fixed *a* and *p* and describe the dynamics of varying the parameter *k*_*a*_. Our simulations show that in the presence of A*β*, changing the parameter *k*_*α*_ can produce aberrant Ca^2+^ signals. These aberrant signals result as transitions through MMOs for fixed A*β* levels. However, we also show that we can control these signals by increasing *k*_2_, the maximal kinetic rate of the RyR.

**Fig 7:**
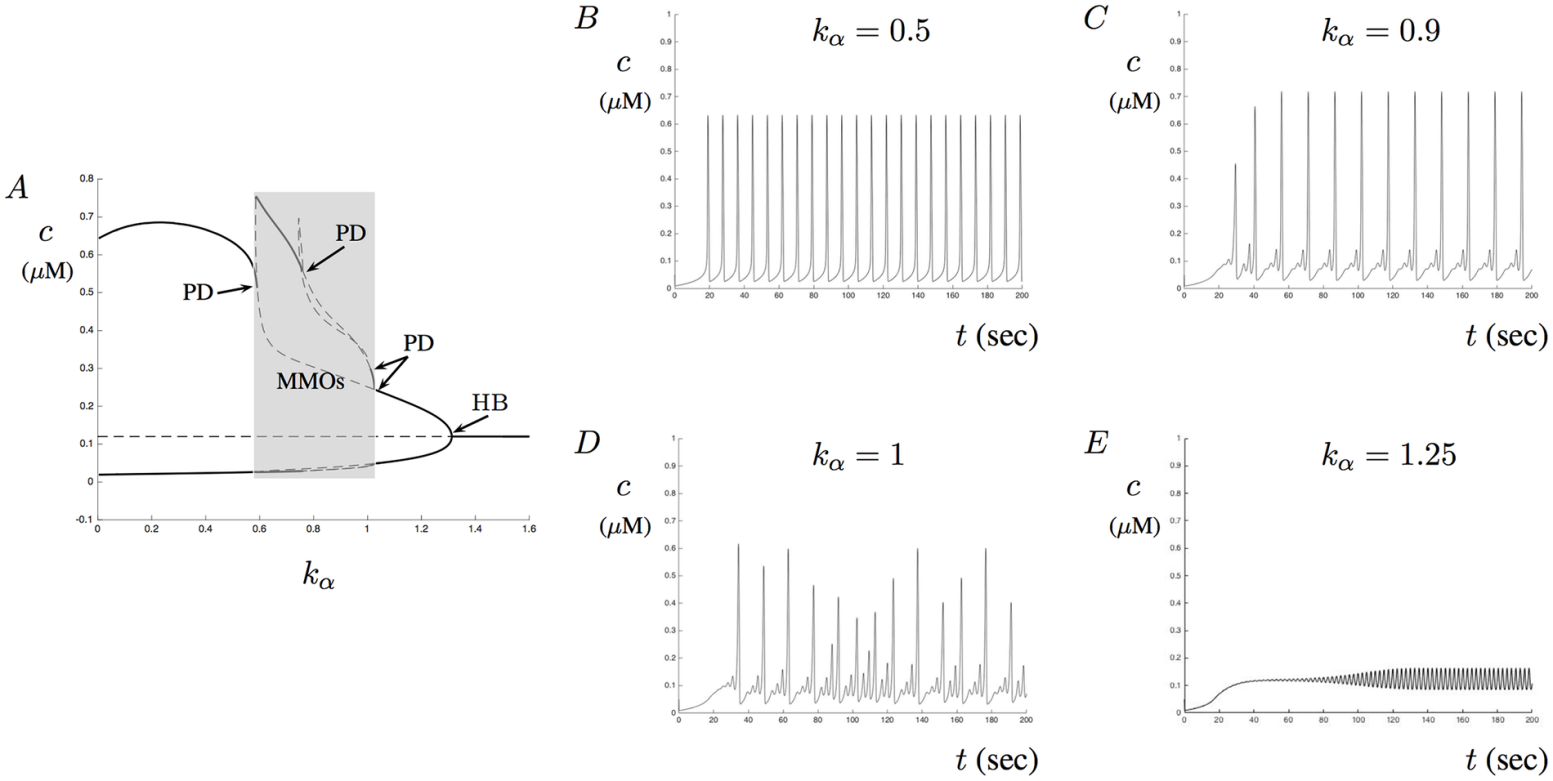
The effects of *k*_*α*_ on Ca^2+^ signals in the presence of A*β*. This figure shows a bifurcation diagram and four Ca^2+^ traces. A shows a partial bifurcation diagram for the parameter *k*_*α*_. In this figure, a number of period doubling points have been labeled as PD. The shaded region corresponds to MMOs. The single Hopf bifurcation point has been labeled with HB. B-E show four solutions for the parameter values *k*_*α*_ = 0.5, 0.9, 1, and 1.25, respectively. D shows aberrant Ca^2+^ signals.

Fig 7 shows four Ca^2+^ traces with *k*_*α*_ = 0.5, 0.9, 1, and 1.25 when *a* = 0.25 and *p* = 10 are fixed. Notice that for *k*_*α*_ = 1 aberrant Ca^2+^ signals emerge. Also included in Fig 7A is the corresponding partial bifurcation diagram for *a* = 0.25 using *k*_*α*_ as the bifurcation parameter. Notice that MMOs exist for a large parameter set of *k*_*α*_. In Fig 7A there are four period doubling bifurcations that each have been labeled as PD. These occur around the parameter values of *k*_*α*_ = {0.5893, 0.7599, 1.01, 1.026}, respectively. The single Hopf bifurcation occurs around the parameter value *k*_*α*_ = 1.313. The bifurcation diagram in A provides us with a way to predict the Ca^2+^ signals for a large set of the parameter *k*_*α*_. Notice that stable periodic solutions occur for the parameter intervals *k*_*αs*_ = (0, 0.5893) ∪ (1.026, 1.313). We have MMOs for the parameter interval *k*_*αmmo*_ = (0.5893, 1.026) with various sub-threshold oscillations patterns. Fig 7D shows aberrant Ca^2+^ signals as the parameter *k*_*α*_ remains close to the period doubling value 1.01. Fig 7E shows small stable periodic oscillations for the value *k*_*α*_ = 1.25 near the Hopf point.

**Fig 8:**
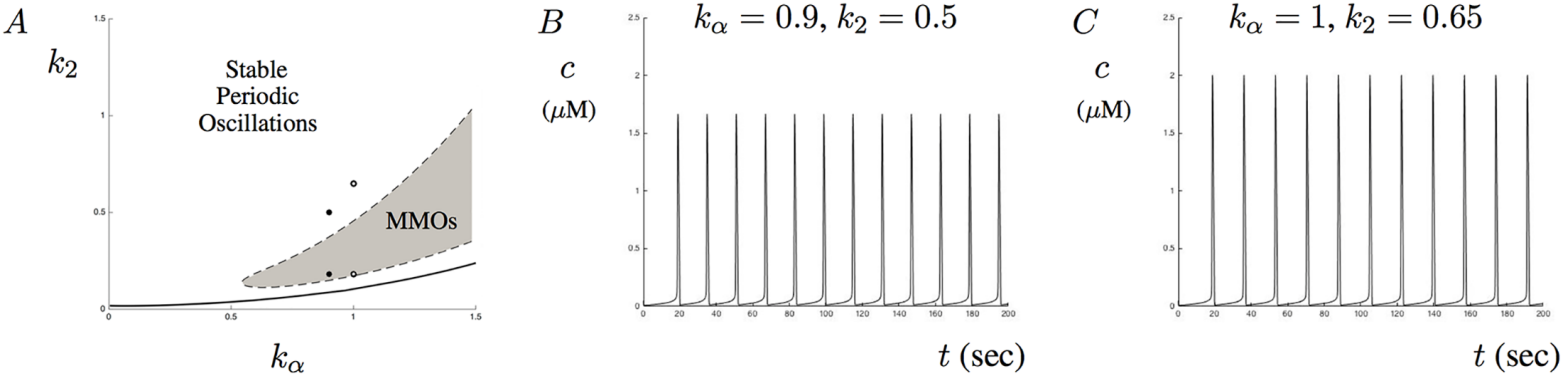
Changing the maximal reaction rate of the RyR. This figures shows the effects of changing the parameter *k*_2_ on model solutions. A shows a two parameter bifurcation diagram with *k*_*α*_ and *k*_2_ as the bifurcation parameters. The shaded region corresponds to the region of MMOs. The solid curve corresponds to the manifold of Hopf bifurcation points. The two dots on the left side represents the locations of the parameter values of *k*_2_ used to generate the Ca^2+^ traces in Fig 7C and Fig 8B. The two dots on the right side represents the locations of the parameter values of *k*_2_ used to generate the Ca^2+^ traces in Fig 7D and Fig 8C. B and C show the stable periodic oscillations that occur when *k*_2_ is increased to *k*_2_ = 0.5 and *k*_2_ = 0.65, respectively.

Each trace illustrated in Fig 7 shows a different type of Ca^2+^ response based on the value of *k*_*α*_. One possible way of dealing with the aberrant signals and MMOs is to drive the maximal reaction rate of the RyR up. This corresponds to increasing the value of *k*_2_ in the model. Fig 8A shows a two-parameter bifurcation diagram with *k*_*α*_ on the *x*-axis and *k*_2_ on the *y*-axis. What this diagram illustrates is the region of MMOs produced by the model for the parameter ranges of *k*_*α*_ and *k*_2_ given in the figure. Suppose that for a fixed *k*_*α*_ value, Ca^2+^ signals undergo aberrant signals or MMOs. By increasing the parameter *k*_2_ sufficiently, passage into stable periodic solutions will occur. This suggests that the maximal rate of the RyR kinetics may help to control both aberrant Ca^2+^ signals and MMOs. To see this, Fig 8B shows the solution corresponding to those in Fig 7C with *k*_*α*_ = 0.9 when *k*_2_ = 0.18 is increased to *k*_2_ = 0.5. Similarly, Fig 8C shows the solution corresponding to those in Fig 7D with *k*_*α*_ = 1 when *k*_2_ = 0.18 is increased to *k*_2_ = 0.65. Notice that although solutions do settle into stable periodic orbits that the amplitude of the signals increase. Thus, increasing the parameter *k*_2_ may help to stabilizes aberrant oscillations at the cost of increasing Ca^2+^ oscillation amplitude. A similar stable region also exists below the region of MMOs. This region would also help control signals but may be more difficult to precisely isolate the appropriate range of *k*_2_.

#### Dynamic levels of IP_3_ with no membrane potential

The model dynamics exhibited in the figures above are triggered by a constant value of IP_3_ in the cytosol. Recall that *in vivo*, IP_3_ production is typically a result of an agonist activated G-couple protein. The rates of IP_3_ production and degradation are both modulated by intracellular Ca^2+^, and as such, the release of Ca^2+^ through the IP_3_ receptor can directly alter the IP_3_ signaling pathway. Furthermore, there is growing evidence that A*β* also affects the IP_3_ signaling pathway [7]. Thus, we extend our model to include a dynamic variable for IP_3_ production and degradation, and look to include the influence of A*β* on this signaling mechanism when *p*_*s*_ = 0. More specifically, we include an additional equation for IP_3_ and model the influence of A*β* on the production of IP_3_.

We make use of the hybrid model formulated by Politi et al. (2006) to track IP_3_ production and degradation. Their model takes the form

$$\tau_{p}\frac{dp}{dt}=V_{PLC}\frac{c^{2}}{K_{PLC}^{2}+c^{2}}-(\frac{\eta c^{2}}{K_{3K}^{2}+c^{2}}+\left(1-\eta \right))p,$$ (20)

where

$$\tau_{p}=\frac{1}{k_{3K}+k_{5P}},\;\text{and}\;\eta =\frac{k_{3K}}{k_{3K}+k_{5P}}.$$

The first term on the right hand side of (20) corresponds to the production of IP_3_ and the second term is the degradation. In (20), *V*_*PLC*_ is the maximal production rate, *K*_*PLC*_ characterizes the sensitivity of PLC, *K*_3*K*_ is the half saturation constant for the degradation term. The constants *k*_3*K*_ and *k*_5*P*_ correspond to the IP_3_ phosphorylation and dephosphorylation rates, respectively. *K*_*PLC*_ and *η* are parameters used to adjust the positive and negative feedback of Ca^2+^, respectively. One of the advantages for using this hybrid model is that it can easily be altered to reproduce both class I and class II mechanisms (see [18] for details). Another advantage, is that this model breaks the IP_3_ process into two components: a production and a degradation. This will make it easier for us to incorporate the effects of A*β* on the IP_3_ production process.

In their experiment, Demuro and Parker (2013) showed that introducing A*β* directly into *Xenopus* oocytes causes an increase in Ca^2+^ dependent fluorescence (a measure for the amount of intracellular Ca^2+^). Even though their experiments are in oocytes, the ubiquitous properties of IP_3_ signaling may make their results relevant to other cells including neurons. Their findings suggest that A*β* does not interact directly with the IP_3_ receptor, but instead they propose that intracellular Ca^2+^ liberation evoked by A*β* involves opening of IP_3_ receptors as a result of stimulated production of IP_3_ via G-protein-mediated activation of PLC. As such, in the presence of A*β*, IP_3_ are actively stimulated and persist for many minutes or hours even though IP_3_ is metabolized within tens of seconds [58]. Based on these findings, we assume that *V*_*PLC*_ takes the form

$$V_{PLC}=v_{PLC}+\mu_{PLC}a,$$ (21)

and *K*_*PLC*_ can be written similarly as

$$K_{PLC}=k_{PLC}+\kappa_{PLC}a,$$ (22)

where the parameters *μ*_*PLC*_ and *κ*_*PLC*_ control the strength of the linear influence of A*β* on each term, respectively. For our purposes, we set both of these values to be *μ*_*PLC*_ = *κ*_*PLC*_ = 1. Thus, we add the following equation to (18) and (19) and look to determine the impact of A*β* on model solutions

$$\tau_{p}\frac{dp}{dt}=\left(v_{PLC}+\mu_{PLC}a\right)\frac{c^{2}}{\left(k_{PLC}+\kappa_{PLC}a\right)+c^{2}}-(\frac{\eta c^{2}}{K_{3K}^{2}+c^{2}}+\left(1-\eta \right))p.$$ (23)

In our simulation we use the work of [59] to set a number of parameter values. However, we assume that both positive and negative feedback are present simultaneously and as such make parameter adjustments as needed. The parameter values that we use for our simulations are given in Table 2. With these additional contributions, we now have a model that includes the impact of A*β* on multiple Ca^2+^ signaling mechanisms. Although the model has a large number of variables and parameters, we seek to characterize model solutions by investigating the dynamical properties of the model in the presence of A*β*.

**Table 2:** Parameter values of the IP_3_ model (23).

IP_3_ parameters					
*v*_*PLC*_	1.5 *μ*M s^−1^	*k*_5*P*_	0.25 s^−1^	*K*_3*K*_	0.4 *μ*M
*k*_*PLC*_	1 *μ*M	*k*_3*K*_	0.5 s^−1^		

**Fig 9:**
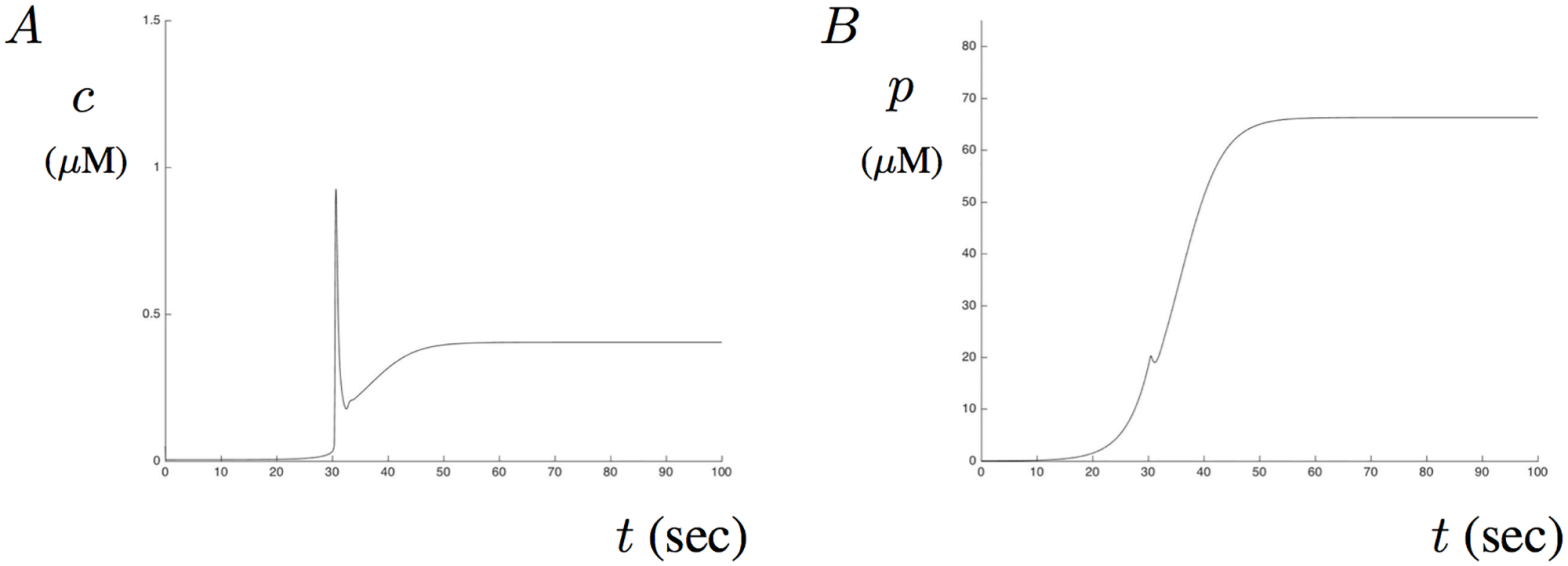
Dynamic IP_3_ without A*β*. A shows Ca^2+^ and B shows IP_3_ as a function of time in the absence of A*β*. A spike of Ca^2+^ occurs once enough IP_3_ has accumulated but does not lead to sustained oscillations. The amount of IP_3_ settles to a steady-state level in B.

In the absence of A*β*, model solution with initial condition for IP_3_ = 0.01 shows a small Ca^2+^ influx followed by a transition to it’s steady-state level close to 0.5 *μ*M. This is consistent with what we expect as the amount of IP_3_ is dynamic and depends on Ca^2+^. It will take sometime for enough IP_3_ to be present in order to trigger a signaling event throught the receptor. Fig 9A and 9B show the model solution for Ca^2+^ and IP_3_, respectively in the absence of A*β*. Notice that the amount of IP_3_ also reaches a steady-state level.

**Fig 10:**
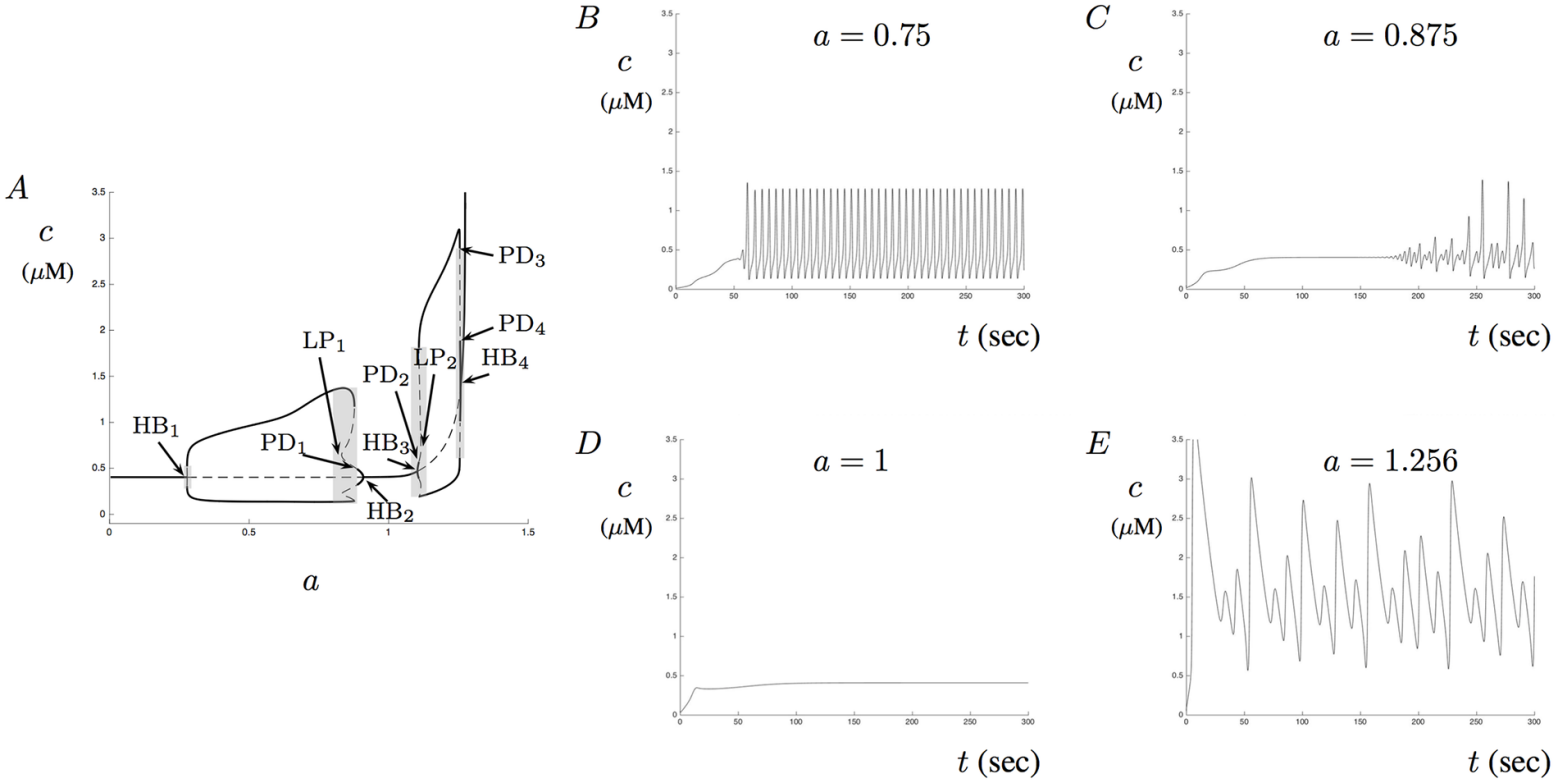
Calcium signals in the presence of A*β* with dynamic IP_3_. This figure shows a bifurcation diagram and four Ca^2+^ traces for the model with dynamic IP_3_. A shows a partial bifurcation diagram where two oscillatory regions are separated by a single steady-state region. The shaded regions near each of the four Hopf bifurcations correspond to MMOs. Numerous period doubling bifurcations occur around the shaded regions. Two important limit points have been labeled LP_1_ and LP_1_. These points are important in the description of solutions. B-D show model solutions for *a* = 0.75, 0.875, 1, and 1.256, respectively. The various patterns in these figures are predicted by the bifurcation diagram in A.

Since we are interested in understanding the potential effect of A*β* on Ca^2+^ signals, we use *a* as a bifurcation parameter and investigate model solutions. Fig 10A shows a partial bifurcation diagram with *a* as the bifurcation parameter. This diagram has two oscillatory regions separated by a region of a single stable-steady state. The four Hopf bifurcations are labeled along with a number of period doubling points. Notice that there are four regions of MMOs (shaded regions) that appear close to each Hopf point. In addition to the Hopf bifurcation points, four period doubling points have also been labeled with two limit points (LP). We provide the value of each of these points in Table 3 and use them to identify solution patterns. As the parameter *a* increases to LP_3_ = 1.274, solutions become unphysical. Thus, we limit our investigation for values of *a* between 0 and 1.274. Fig 10B–10D show Ca^2+^ traces predicted by Fig 10A for various values of *a*. Notice that aberrant signals are also present for the model with a dynamic equation for IP_3_.

**Table 3:** Solution behavior for model with dynamic IP_3_.

Parameter *a* interval	Point type	Description of solution pattern
(0, 0.2783)	(0, HB_1_)	Stable steady-state solutions
(0.2783, 0.8318)	(HB_1_, LP_1_)	Stable periodic solutions
(0.8318, 0.8857)	(LP_1_, PD_1_)	Mixed-mode oscillations
(0.8857, 0.909)	(PD_1_, HB_2_)	Stable periodic solutions with small amplitude
(0.909, 1.103)	(HB_2_, HB_3_)	Stable steady-state solutions
(1.103, 1.109)	(HB_3_, PD_2_)	Stable periodic solutions with small amplitude
(1.109, 1.16)	(PD_2_, LP_2_)	Mixed-mode oscillations
(1.16, 1.255)	(LP_2_, PD_3_)	Stable periodic solutions with large amplitude
(1.255, 1.256)	(PD_3_, PD_4_)	Mixed-mode oscillations with elevated Ca^2+^ levels
(1.256, 1.257)	(PD_4_, HB_4_)	Stable periodic solutions with small amplitude but with elevated Ca^2+^ levels
(1.257, 1.274)	(HB_4_, LP_3_)	Stable high Ca^2+^ steady-state solutions

### Calcium model with influence of membrane potential

**Fig 11:**
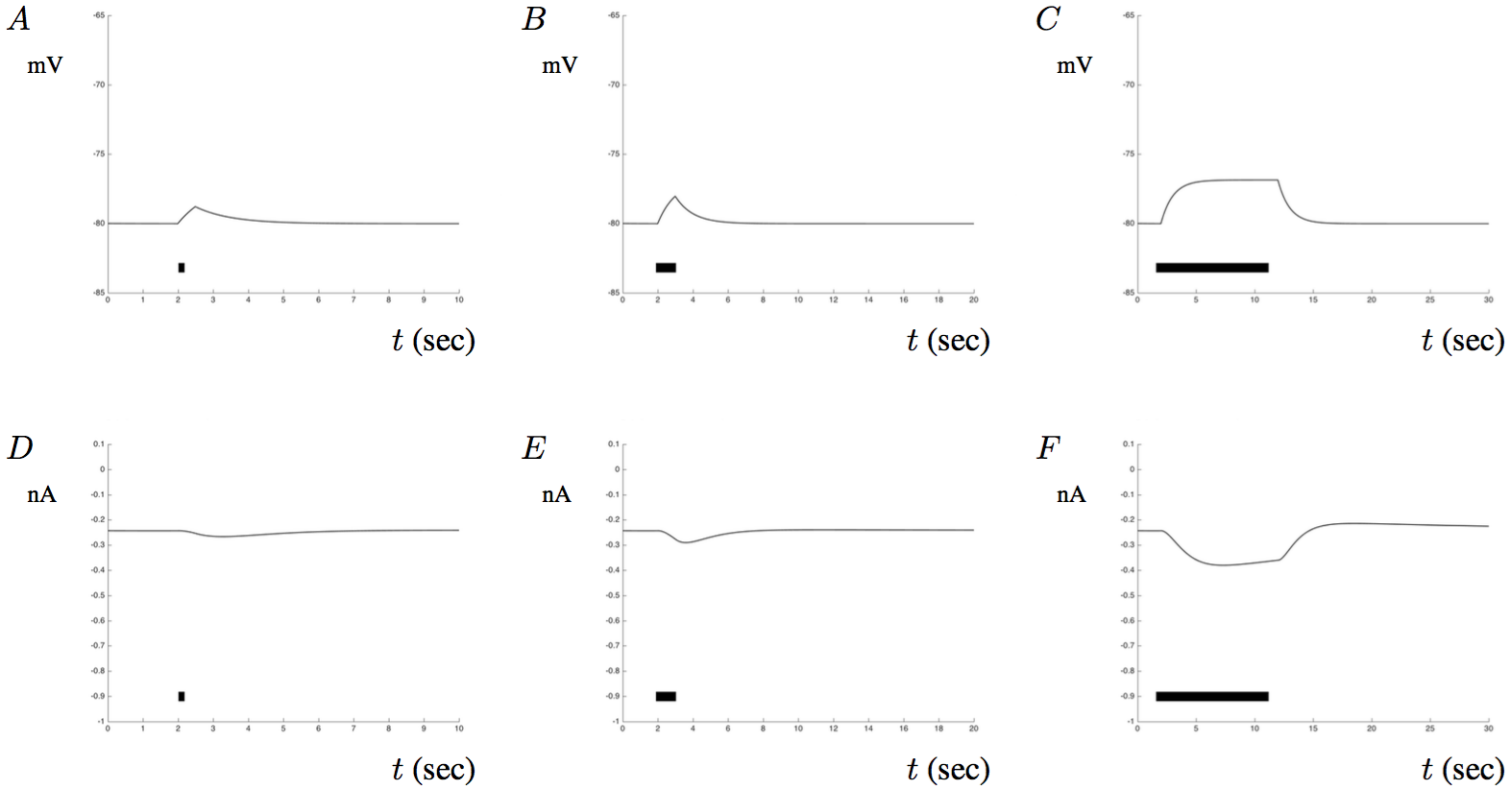
Ca^2+^ flux due to changes in membrane potential. This figures shows the Ca^2+^ current *I*_*ca*_ in response to changes in membrane potential. A-C show changes in the membrane potential of the astrocytic model when a current of 300 nA is applied for a duration of 0.1 second (A), 1 second (B), and 10 seconds (C). The current was applied at *t* = 2 seconds and is represented by the black bar at the bottom of each figure. D-F show the corresponding VGCC current *I*_*ca*_ as defined by (15).

In our simulations below, we examine the effects of changes to membrane potentials by first incorporating the membrane into the model and by stimulating the membrane with a constant applied current pulse. The impact of such an applied current on the voltage *V* and the resulting Ca^2+^ current are shown in Fig 11. A stimulating current of 300 nA applied for *t* = 0.1 seconds initiated at *t* = 2 generates the potential response shown in Fig 11A. Fig 11B and 11C show the response due to an applied stimulus lasting for one and ten seconds, respectively. These changes in membrane potential cause the inward currents through the VGCC illustrated in Fig 11D–11F, for *t* = 0.1, *t* = 1, and *t* = 10 seconds, respectively. Notice that in Fig 11C the membrane voltage saturates as the duration of the applied current is increased. Although the mechanisms for generating these signals is fairly simplistic, our results align well with experimental data showing similar saturating levels in astrocytes [38]. The effects of the inward Ca^2+^ through the VGCC are included in the flux term *J*_*vca*_ and as such, allows us to study the impact of membrane potentials on Ca^2+^ signals. In all subsequent figures, we have applied a constant current pulse at *t* = 100 for a duration of 50 seconds. Such an applied current does not capture an *in vivo*-like representation of membrane potentials, but it does offer a way to link the model with typical voltage clamp experiments where the amplitude and duration of the applied current can be controlled.

**Fig 12:**
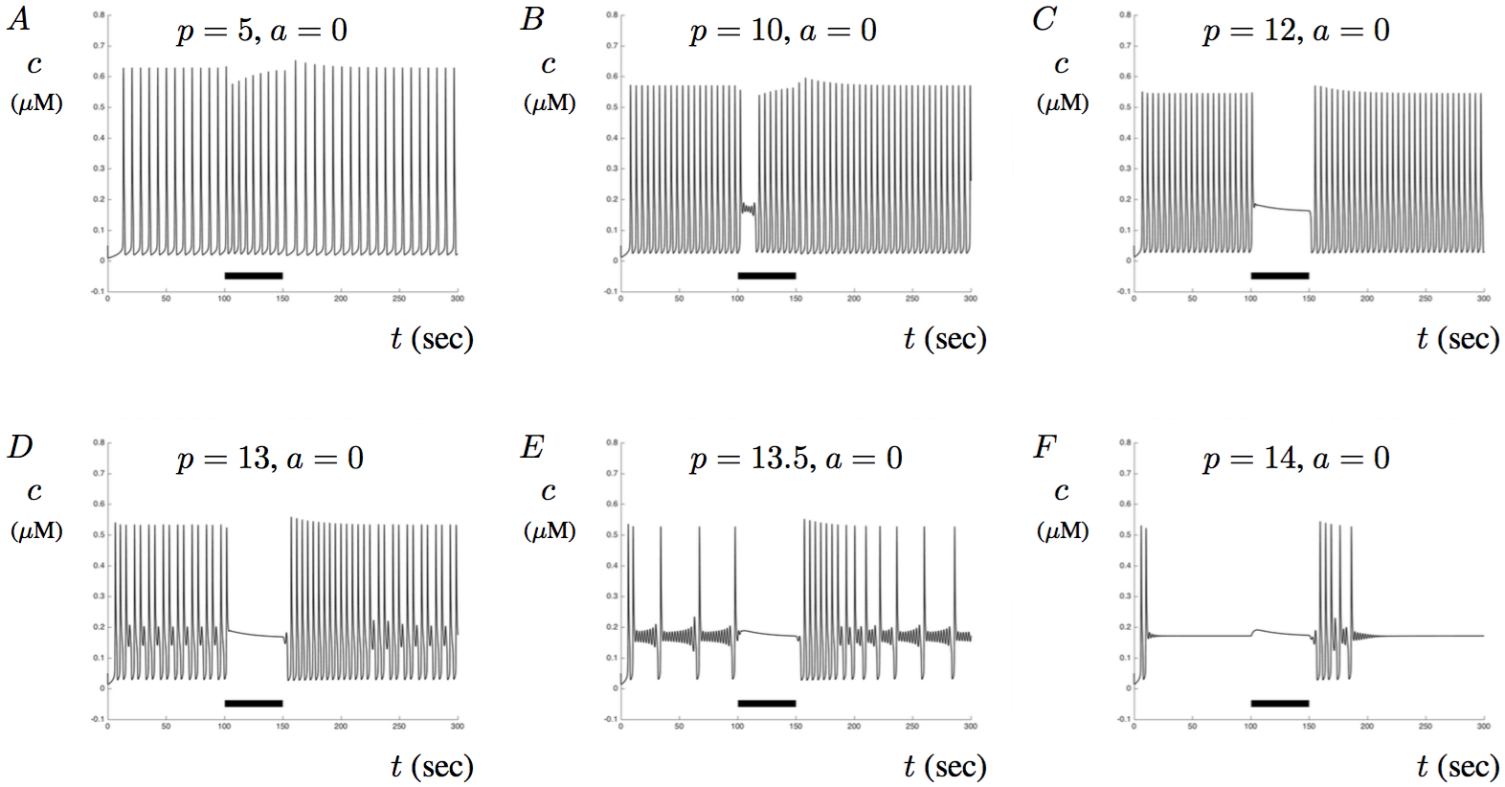
The influence of IP_3_ on Ca^2+^ signals with membrane potential. This figures shows the impact of membrane potential on Ca^2+^ signaling. In each figure, membrane potentials are included as a response to a sustained applied current of 300 nA lasting from *t* = 100 to *t* = 150. Figs A, B, and C show intracellular Ca^2+^ signals when *p* = 5, *p* = 10, and *p* = 12 with *a* = 0, respectively. Figs D, E, and F show the response when *p* = 13, *p* = 13.5, and *p* = 14, respectively. Note that the inclusion of the membrane potential filters MMOs and can help establish transient single-mode oscillations upon the termination of the applied current (D,E).

To investigate the impact of a constant current pulse on Ca^2+^ signaling, we first simulate (18) and (19) with constant values of IP_3_. In these simulations we set *p*_*s*_ = 1 and plot Ca^2+^ concentrations as a function of time. Tracking the membrane potential and including it into the model will have an effect on Ca^2+^ signals. Specifically, the membrane will alter the dynamics of the effect of *p* on model solutions. In order to illustrate this point we have plotted the model responses when no A*β* is present for various values of *p* in Fig 12. When these solutions are compared to Fig 2, we can see that the inclusion of the membrane potential increases the response frequency and MMOs occur for smaller values of *p* (for example *p* = 13 here instead of *p* = 17 in Fig 2).

Notice that in Fig 12D–12F Ca^2+^ signals seem to stabilize in single-mode oscillations upon the release of the stimulus before transitioning back into MMOs. This may provide one possible path for stabilizing Ca^2+^ signaling. In an *in-vivo* like environment, membrane potentials vary based on intrinsic response mechanisms to a stimulus. Our results show that artificially triggering membrane stimulation could potentially help to stabilize Ca^2+^ signals. Furthermore, Fig 12E shows that the model solution can exhibit a large number of sub-threshold oscillations before triggering a larger spike. This type of MMO is different from those illustrated previously and shows that membrane potentials can have an impact on global Ca^2+^ signals and play an important role in Ca^2+^ regulation. A full analysis for understanding these transitions is beyond the scope of this work but could prove useful for predicting how membrane stimulation may be used to control and/or stabilize aberrant Ca^2+^ signals.

**Fig 13:**
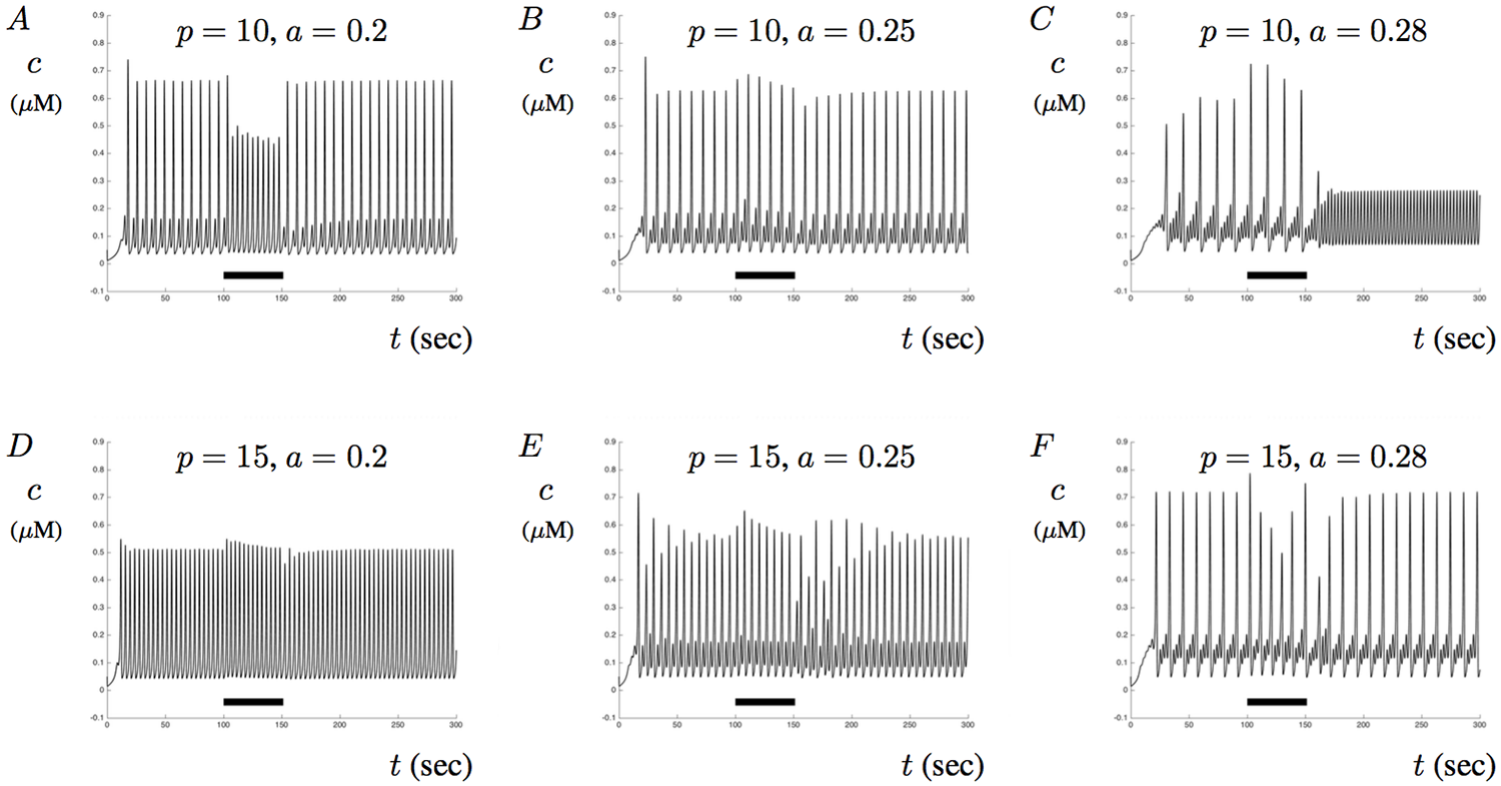
The influence of A*β* on Ca^2+^ signals with membrane potential. This figures shows the impact of membrane potential on Ca^2+^ signaling. In each figure, changes in membrane potential are included as a response to a sustained applied current of 300 lasting from *t* = 100 to *t* = 150. Figs A, B, and C show intracellular Ca^2+^ signals when *p* = 10 and when *a* = 0.2, *a* = 0.25, and *a* = 0.28, respectively. Figs D, E, and F show the response when *p* = 15 and when *a* = 0.2, *a* = 0.25, and *a* = 0.28, respectively.

Model solution patterns are not only linked to the amount of IP_3_ and the inclusion of membrane potential, but also by the amount of A*β* present in the model. To better understand the roles of IP_3_, membrane potentials, and A*β* on Ca^2+^ signaling, we simulate the model and provide the solutions when *p* = 10 and *p* = 15 with various levels of A*β* for *p*_*s*_ = 1. Illustrated in Fig 13 are six model solutions that illustrate the impact of A*β* and membrane potentials on Ca^2+^ signaling. When *p* = 10 is fixed and *a* is altered, model behavior is directly impacted by the application and removal of a constant current pulse. This is illustrated in Fig 13A–13C where *a* = 0.2, *a* = 0.25, and *a* = 0.28. Specifically, Fig 13C shows that upon the termination of the applied stimulus, Ca^2+^ signals do not go back to MMO patterns but instead transition to stable single-mode oscillations. This shows that stimulation of the membrane can alter intrinsic dynamical patterns and be used to stabilize various types of Ca^2+^ signals. Fig 13D–13F show model solutions for *p* = 15 with *a* = 0.2, *a* = 0.25, and *a* = 0.28, respectively. It is interesting to note that when we apply a constant current pulse, Ca^2+^ solutions can transition into steady MMOs as the level of A*β* increased towards *a* = 0.28. Although we do not analyze the bifurcation structure, the inclusion of membrane potentials in the model appears to have altered the parameter dependance where regions of MMOs can occur as well as transitions from single- to mixed-mode oscillations. Further analysis may be beneficial for drawing out the underlying mechanisms in the stable oscillatory patterns when a constant current is applied in the model.

**Fig 14:**
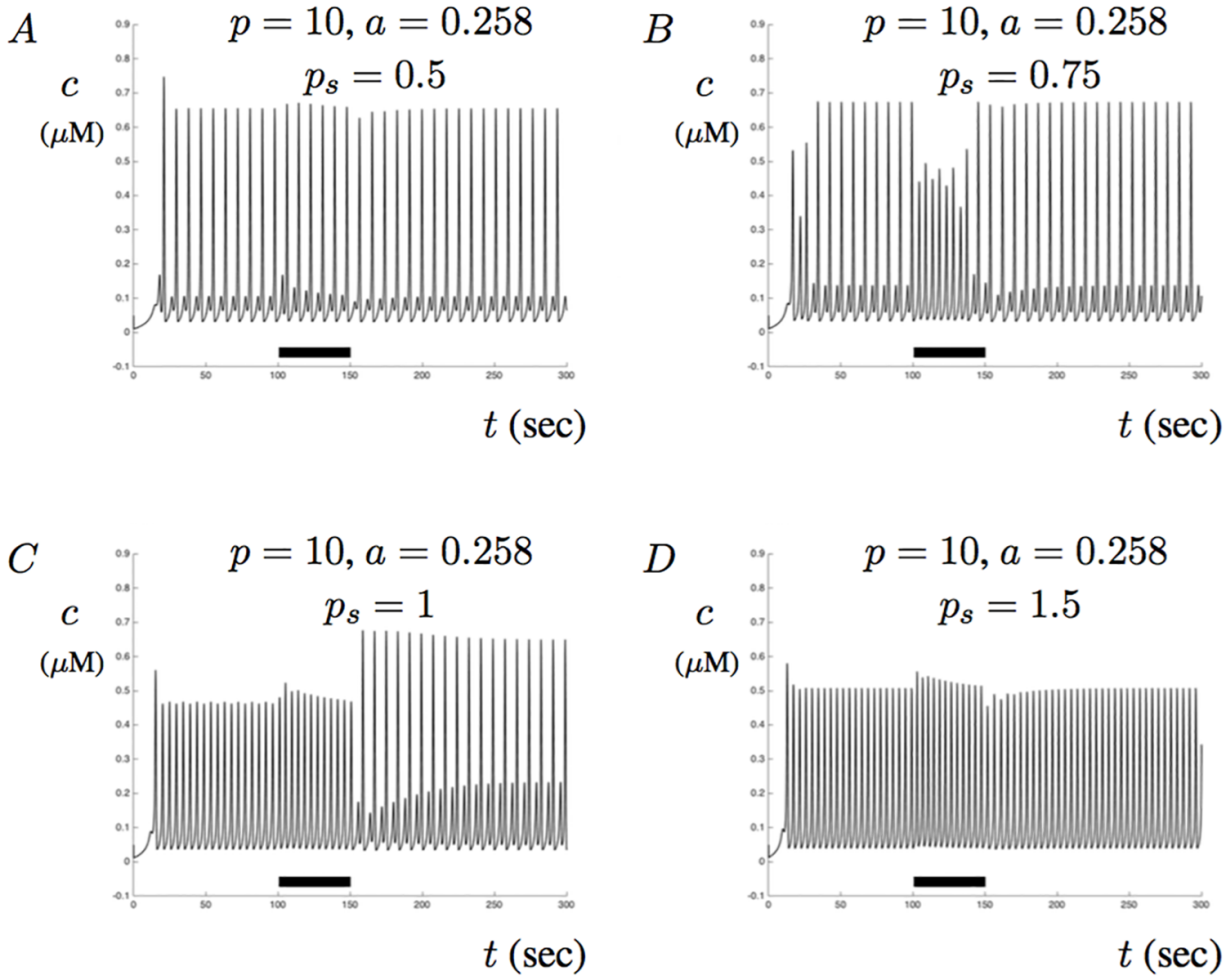
The impact of scaling *I*_*ca*_ on model solutions. This figures shows various model solutions when the scaling parameter *p*_*s*_ is altered. In each figure, *p* = 10, and the amount of A*β* is fixed at *a* = 0.258. A-D show the response of (18) and (19) when *p*_*s*_ = 0.5, *p*_*s*_ = 0.75, *p*_*s*_ = 1, and *p*_*s*_ = 1.5, respectively. Notice that B shows that Ca^2+^ can enter aberrant oscillatory patterns when the membrane is stimulated by a constant applied current. C shows that Ca^2+^ signals can enter MMOs with altered amplitudes when the applied current stimulus is turned off.

Because the impact of membrane potentials are controlled by *p*_*s*_, we have also provided model simulations when this parameter is altered. Small values of *p*_*s*_ correspond to small influence of membrane potential while larger values can be used drive the amplitude of Ca^2+^ signals. Fig 14 shows four simulations for four different values of *p*_*s*_. When *p*_*s*_ = 0.5, Fig 14A shows that the effects of membrane potentials are small and no significant changes in Ca^2+^ are observed other than a slight increase in the subtreshold oscillations. When *p*_*s*_ = 0.75, Fig 14B suggests that the effects of membrane potentials are large enough to alter the amplitude of Ca^2+^ oscillations during the applied current. Fig 14C shows that when *p* = 1, contributions from membrane potentials increase overall Ca^2+^ signaling amplitude. With these values of *p* and *a*, the solution transitions from single mode oscillations with amplitude around 0.45 to MMO with an increased amplitude to around 0.7. Fig 14D shows that a large influence of membrane potentials (when *p* = 1.5) does not alter the signal amplitude or oscillatory mode significantly.

Although much analysis remains to fully understand the dynamics of the model, the results of our simulations suggest that A*β* can alter Ca^2+^ regulatory mechanisms in a way that leads to both MMOs and aberrant signaling. We have shown that bifurcation regions for dynamical transitions from single mode to MMOs can increase in the presence of A*β*. By altering RyR receptor dynamics, we show that transitions from MMO back to single-mode oscillations can occur. Furthermore, we show that stimulation of the membrane can also be used to control various types of Ca^2+^ signals. Although we have made a number of simplifying assumptions in the model development, our approach can be easily altered to include other more complex interactions and mechanisms that influence Ca^2+^ regulation.

## Discussion

Intracellular Ca^2+^ is a critically important second messenger within the nervous system. In neurons, Ca^2+^ is known to mediate the signaling pathways that control neurotransmitter release, gene expression, metabolism, plasticity, development, proliferation, and cell death [60]. As such, Ca^2+^ may play a major role in the pathogenesis of AD. Unfortunately, the complexity of Ca^2+^ signaling makes it difficult to precisely understand how A*β* impacts different intracellular regulation mechanisms and components. Various studies have decoupled particular components and merging these theories together to form a whole-cell computational model can help us better understand intracellular Ca^2+^ regulation and what leads to aberrant signaling. One of our goals is to model Ca^2+^ in a simplified whole-cell environment that has predictable qualitative structure so that we can study the effects of A*β* on the dynamics. As such, the qualitative features described in this study give us a way to track Ca^2+^ patterns using dynamical systems theory.

The simulations presented in this study occur on the order of seconds to minutes while the progression of AD occurs on the timescale of months to years. However, in our model we are using the accumulation of A*β* to describe the potential stage in the evolution of AD. Even before toxic A*β* plaques can aggregate, the slow accumulation of A*β* peptides can trigger alterations in Ca^2+^ signaling patterns. Our model shows that aberrant signals and changes in homeostasis levels can emerge as the amount of A*β* is increased. In an *in vivo* environment these changes may be subtle and actually evolve over days or months. Any alterations in intracellular Ca^2+^ homeostasis can affect the apoptotic signaling cascade. Both the mitochondria and the ER play a significant role in apoptosis and are sensitive to changes in Ca^2+^ levels. Although we have not considered mitochondrial effects, we do track ER Ca^2+^. Further analysis that looks at the time evolution of *c*_*e*_ could be useful in predicting chronic changes of Ca^2+^ homeostasis in AD.

Developing a whole-cell Ca^2+^ model that has predictable qualitative structure in the presence of A*β* is challenging. Although A*β* influences many Ca^2+^ regulatory mechanisms, the particular way which A*β* affects these mechanisms is generally not known. Additionally, the temporal influence of A*β* on certain mechanisms could occur on the order of milliseconds, seconds, days, months, or years. As such, any computational model will necessarily make a number of simplifying assumptions. Even by exploiting these simplifications, our model includes a large number of parameters that make mathematical analysis limited. Unfortunately, we do not have robust estimates for many of the parameters involved in the model. However, we have attempted to provide justification for many assumptions and parameter choices based on the literature and the experimental data currently available. We do recognize that many of these assumptions may need to be altered as we continue to improve our understanding of the effects of A*β* in an AD environment.

The ubiquitous nature of the Ca^2+^ regulatory mechanisms used in our model makes it easily adaptable for studying various cell types with spatial components. Specifically, A*β* has been shown to cause complex Ca^2+^ signals in astrocytes [61, 62]. In these astrocytes, Ca^2+^ waves and oscillations signals can occur on timescales even slower than those typical of other non-excitable neuroglia. Although our modeling approach does not include spatial components, additional mechanisms can be constructed to account for wave generating behaviors. Furthermore, astrocytes can facilitate synaptic transmission and plasticity through the uptake of neurotransmitter [63] and complex models between neurons and astrocytes have been developed to study these interactions [38, 64, 65]. Both microglia and astrocytes have been described as modulators for A*β* clearance and degradation [66] and our approach may be useful for better understanding these mechanisms.

It is clear that A*β* plays an essential role in the cognitive decline in AD by directly affecting synaptic transmission [11, 67–70]. However, synaptic transmission is typically precipitated by a presynaptic potential which allows Ca^2+^ ions to flow into the cell through VGCC. The contributions of fast local Ca^2+^ signals with slow global Ca^2+^ patterns, especially under the influence of A*β*, may help explain why breakdowns in synaptic efficacy can occur in an AD environment [11, 45, 67, 69, 71, 72]. The accumulation or presence of A*β* may directly, or indirectly, impact various Ca^2+^ driven mechanisms during synaptic transmission. The simplified whole-cell Ca^2+^ model presented here could be linked with a synaptic Ca^2+^ model to investigate how global aberrant Ca^2+^ signals may impact synaptic transmission on multiple timescales. Simulations over long timescales may help explain how slow global whole-cell Ca^2+^ signals interfere with fast local Ca^2+^ signals at the synapse.

Different individual models exist for the various signaling components used in our simplified whole-cell model development. For example, we used the Sneyd and Dufour (2002) formulation for the IP_3_ receptor model. Although this model is sound and well-suited for our purposes, it does increase the number of necessary variables considerably. One could use a two equation model for IP_3_ (such as those described in [26, 73]), but the number of parameters will remain large. Similarly, alternative models for the RyR may tease out alternative conclusions when influenced by A*β* (such as using a model as in [74]). As such, we encourage further development of the model as experimental data becomes available. Matching model dynamics with experimentally recorded data can help select the component model best suited for the particular study.

Our model solutions are highly sensitive to certain parameters and the oscillatory responses presented here only occur under certain scenarios. Because of the complexity of Ca^2+^ regulation along with understanding the impact of A*β*, any computational model would benefit from both local and global sensitivity analysis. Although we have not performed any sensitivity analysis, we do understand that much of the analysis and many of our conclusions may be valid for a small set of parameters. Further, the sensitivity of a particular parameter may influence how the model transitions into aberrant Ca^2+^ signals. As such, we recommend that sensitivity analysis be performed as a next step in order to better understand the role of parameters on model dynamics.

In conclusion, we have shown that aberrant Ca^2+^ signals can occur in a simplified whole-cell model under the influence of A*β*. Furthermore, we showed that regions of MMOs can expand as a consequence of increasing the amount of A*β* in the model. This may partially explain how Ca^2+^ signals are impacted by A*β* from a dynamics perspective within an *in vivo* like environment. Continued refinement of the model in conjunction with experimental data matching will help make the model more useful. In turn, this can help us determine how to control for both aberrant signals and increased homeostasis Ca^2+^ levels. The model can then be used to better understand the impact of A*β* on Ca^2+^ fluxes through individual regulatory components (such as IP_3_, RyR, and plasma membrane). This computational model can help us study complex cellular behavior in an AD environment by tracking the influence of many interconnected biological mechanisms.

## Supporting information

S1 Appendix A summary of both the IP_3_ receptor model equations and those governing the membrane potential. Also provided in the appendix are the model parameters used in the Hodgkin and Huxley formulation of the membrane potential.
(PDF)
