# Supplementary material for: A mathematical model for the effects of amyloid beta on intracellular calcium
Source: PLoS One. 2018 Aug 22;13(8):e0202503. doi: 10.1371/journal.pone.0202503 (PMC6105003; doi:10.1371/journal.pone.0202503)
Supplement: S1 Appendix — Also provided in the appendix are the model parameters used in the Hodgkin and Huxley formulation of the membrane potential. (PDF) [file pone.0202503.s001.pdf]

Supplimentary Information:

A mathematical model for the effects of amyloid beta on intracellular calcium

Joe Latulippe[1*], Derek Lotito [2], Donovan Murby[1]

1 Mathematics Department, Norwich University, Northfield, Vermont, USA
2 Chemistry and Biochemistry Department, Norwich University, Northfield, Vermont, USA

S1 Appendix

Here we provide a summary of both the IP_3 receptor model equations and those governing the membrane potential. We also give the model parameters used in the Hodgkin and Huxley formulation of the membrane potential.

The IP_3 receptor has six states that can be reduced to the following five equations

$$\frac{dR}{dt} = \phi_{-2}O - \phi_2 pR + k_{-1}I_1 - \phi_1 R, \tag{1}$$

$$\frac{dO}{dt} = \phi_2 pR - (\phi_{-2} + \phi_4 + \phi_3)O + \phi_{-4}A + k_{-3}S, \tag{2}$$

$$\frac{dA}{dt} = \phi_4 O - \phi_{-4}A - \phi_5 A + k_{-1}I_2, \tag{3}$$

$$\frac{dI_1}{dt} = \phi_1 R - k_{-1}I_1, \tag{4}$$

$$\frac{dI_2}{dt} = \phi_5 A - k_{-1}I_2, \tag{5}$$

where $R + O + A + S + I_1 + I_2 = 1$, and where each rate function $\phi(c)$ between states is given below. The rate functions for the IP_3 receptor model are taken from [1] which are a simplification of the Sneyd and Dufour (2002) model. The rate functions are given by

$$\phi_1(c) = \frac{\alpha_1 c}{\beta_1 + c} \tag{6}$$

$$\phi_2(c) = \frac{\alpha_2 + \beta_2 c}{\beta_1 + c} \tag{7}$$

$$\phi_3(c) = \frac{\alpha_3}{\beta_3 + c} \tag{8}$$

$$\phi_4(c) = \frac{\alpha_4 c}{\beta_3 + c} \tag{9}$$

$$\phi_5(c) = \frac{\alpha_5 c}{\beta_5 + c} \tag{10}$$

$$\phi_{-2}(c) = \frac{\alpha_{-2} + \beta_{-2} c}{\beta_3 + c} \tag{11}$$

$$\phi_{-4}(c) = \frac{\alpha_{-4}}{\beta_5 + c} \tag{12}$$

The relevant parameters for the IP_3 receptor model used in the simulations are provided in [2]. This model was chosen since it does respond reasonably well to changes in Ca^{2+} and IP_3 [3].

The formulation of our Hodgkin and Huxley like membrane potential is given by

$$C_m \frac{dV}{d\tilde{t}} = -I_{kir}(V) - \bar{g}_{na} m^3 h (V - V_{na}) - \bar{g}_l (V - V_l) - I_{ca}(V) + I_{app}, \quad (13)$$

$$\frac{dm}{dt} = \alpha_m(V)(1-m) - \beta_m(V)m, \quad (14)$$

$$\frac{dh}{dt} = \alpha_h(V)(1-h) - \beta_h(V)h, \quad (15)$$

where $C_m = 15$ μF/cm^2, $\bar{g}_{na} = 120$ mS/cm^3, $\bar{g}_l = 0.1$ mS/cm^3, $V_{na} = 50$ mV, $V_l = -74$ mV, with the following gating functions

$$\alpha_m(V) = \frac{0.1\,(V+40)}{(1-\exp(-(V+40)/10))}, \quad (16)$$

$$\beta_m(V) = 4\exp(-(V+65)/18), \quad (17)$$

$$\alpha_h(V) = 0.07\exp(-(V+65)/20), \quad (18)$$

$$\beta_h(V) = \frac{1}{(1+\exp(-(V+35)/10))}. \quad (19)$$

The calcium current in (13) takes the form

$$I_{ca}(V) = \bar{g}_{caT} m_{caT}^2(V) h_{caT}(V)(V - V_{ca}), \quad (20)$$

where the gating activation $m_{caT}(V)$ and inactivation $h_{caT}(V)$ have the form

$$\frac{dm_{caT}}{d\tilde{t}} = \frac{m_{caT,\infty}(V) - m_{caT}}{\tau_{m_{caT}}(V)}, \quad (21)$$

and

$$\frac{dh_{caT}}{d\tilde{t}} = \frac{h_{caT,\infty}(V) - h_{caT}}{\tau_{h_{caT}}(V)}, \quad (22)$$

with

$$m_{caT,\infty}(V) = \frac{1}{(1+\exp(-(V+56.1)/10))}, \quad (23)$$

$$\tau_{m_{caT}}(V) = \frac{1}{(1+\exp((V+86.4)/4.7))}, \quad (24)$$

$$h_{caT,\infty}(V) = \frac{7}{(\exp((V+50)/9)+\exp(-(V+50)/9))} + 0.8, \quad (25)$$

$$\tau_{h_{caT}}(V) = 22. \quad (26)$$

The form and values of the gating variables were chosen to match those used by LeBeau et al. (2000) in their study using hypothalamic neurons [4]. Also note that in our simulations involving membrane potentials, we used a timescale

$$\tilde{t} = C_m\left(1 + \exp\left(\frac{V - V_{ka} - V_{a2}}{V_{a3}}\right)\right)/(g_{kir}\sqrt{K_0}) \quad (27)$$

as in [5] to account for the rectifier channel-mediated return to equilibrium of the astroglial membrane potential.

References

[1] Keener J, Sneyd J. Mathematical Physiology I: Cellular Physiology. 2nd ed. Springer Science+Business Media, LLC; 2009.

[2] Sneyd J, Dufour JF. A dynamic model of the type-2 inositol trisphosphate receptor. Proc Natl Acad Sci U S A. 2002;99(4):2398–403. doi:10.1073/pnas.032281999.

[3] Sneyd J, Tsaneva-Atanasova K, Bruce JI, Straub SV, Giovannucci DR, Yule DI. A model of calcium waves in pancreatic and parotid acinar cells. Biophys J. 2003;85(3):1392–405. doi:10.1016/S0006-3495(03)74572-X.

[4] LeBeau AP, Van Goor F, Stojilkovic SS, Sherman A. Modeling of membrane excitability in gonadotropin-releasing hormone-secreting hypothalamic neurons regulated by Ca2+-mobilizing and adenylyl cyclase-coupled receptors. J Neurosci. 2000;20(24):9290–7.

[5] Sibille J, Dao Duc K, Holcman D, Rouach N. The neuroglial potassium cycle during neurotransmission: role of Kir4.1 channels. PLoS Comput Biol. 2015;11(3):e1004137. doi:10.1371/journal.pcbi.1004137.
